# On four epibiotic peritrichous ciliates (Protozoa, Ciliophora) found in Lake Weishan Wetland: morphological and molecular data support the establishment of a new genus, *Parapiosoma* gen. nov., and two new species

**DOI:** 10.1007/s42995-023-00184-z

**Published:** 2023-08-22

**Authors:** Tong Wu, Ting Cheng, Xiao Cao, Yaohan Jiang, Khaled A. S. Al-Rasheid, Alan Warren, Zhe Wang, Borong Lu

**Affiliations:** 1 Marine College, Shandong University, Weihai, 264209 China; 2 Institute of Evolution and Marine Biodiversity, Ocean University of China, Qingdao, 266003 China; 3 Zoology Department, College of Science, King Saud University, Riyadh, 11451 Saudi Arabia; 4 Department of Life Sciences, Natural History Museum, London, SW7 5BD UK; 5 Weishan Fishery Development Service Center, Jining, 277600 China

**Keywords:** Ectoparasite, *Parapiosoma* gen. nov., Sessilid peritrichs, Systematics

## Abstract

During a study on the diversity of ciliated protists in Lake Weishan Wetland, the largest wetland in northern China, four epibiotic sessilid peritrichs were isolated from aquatic host animals. Two of them, i.e., *Epistylis cambari* Kellicott, 1885 and *Epistylis lwoffi* Fauré-Fremiet, 1943, were known species whereas the other two, i.e., *Parapiosoma typicum* gen. nov., sp. nov. and *Orborhabdostyla gracilis* sp. nov., are new to science. The new genus *Parapiosoma* gen. nov. is characterized by its branched non-contractile stalk, everted peristomial lip, obconical macronucleus and transverse silverlines. Two species are assigned to the new genus, namely *Parapiosoma typicum* sp. nov. and *Parapiosoma gasterostei* (Fauré-Fremiet, 1905) comb. nov. Morphologically, *P. typicum* sp. nov. is recognized by its goblet-shaped zooids, single-layered peristomial lip, dichotomously branched stalk, and infundibular polykinety 3 (P3) containing three equal-length rows. *Orborhabdostyla gracilis* sp. nov. is characterized by its slender zooid, curved macronucleus, and three equal-length rows in infundibular P3. Improved diagnoses and redescriptions of *E. cambari* and *E. lwoffi* are provided including, for the first time, data on the ciliature of *E. cambari*. Phylogenetic analyses based on SSU rDNA, ITS1-5.8S rDNA -ITS2, and LSU rDNA sequence data strongly support the assertion that the family Epistylididae comprises morphospecies with different evolutionary lineages and indicate that *Parapiosoma* gen. nov. may represent a new taxon at family level.

## Introduction

Ciliated protists (ciliates) are a group of unicellular eukaryotes with high species diversity that are an important component of aquatic ecosystems (Campello-Nunes et al. 2022; Chen et al. 2022; Chi et al. 2021, 2022; Ma et al. 2022a, b; Méndez-Sánchez et al. 2022; Omar et al. 2022; Safi et al. 2022; Song et al. 2022; Wang et al. 2022b; Wu et al. 2020; Ye et al. 2022; Zhang et al. 2022a, b). The order Sessilida Kahl, 1933, which comprises about 105–140 genera and more than 800 species, is widely distributed in aquatic environments and has attracted the intertest of researchers for almost 350 years (Foissner et al. 1992, 2010; Kahl 1935; Lynn 2008; Nenninger 1948; Stiller 1971). In recent decades, researchers have been investigating new ways to identify species, e.g., using silver staining methods, and exploring their molecular systematics, mostly using ribosomal DNA (rDNA) gene sequence data (Li et al. 2008a; Liao et al. 2021; Miao et al. 2004; Sun et al. 2016). As more rDNA sequences of sessilids have become available, the classifications of some families have been queried (Lu et al. 2023; Wang et al. 2022c).

One of the most problematic sessilid families is Epistylididae Kahl, 1933, which is mainly characterized by the strongly everted peristomial lip and the non-contractile stalk (Gentekaki et al. 2017; Jiang et al. 2019). In recent years, new genera and species of Epistylididae have been continuously reported, suggesting that there is a large, undiscovered diversity of this family (Canals and Salvadó i Cabré 2016; Kühner et al. 2016; Wang et al. 2021, 2022d). Lu et al. (2023) raised two epistylidid genera, i.e., *Campanella* and *Rhabdostyla*, to family level based on a combination of morphological and molecular data. However, the remaining epistylidids still do not form a monophyletic group and their systematics remains confused due to the lack of sufficient morphological and molecular data for most species. Additionally, recent studies have shown that epibiotic epistylidids could provide new insights for exploring the evolutionary routes for sessilids, although compared to their free-living counterparts, investigations of epibiotic epistylidids are limited (Lu et al. 2020; Song et al. 2003; Wang et al. 2017; Zhou et al. 2019a, b).

**Fig. 1:**
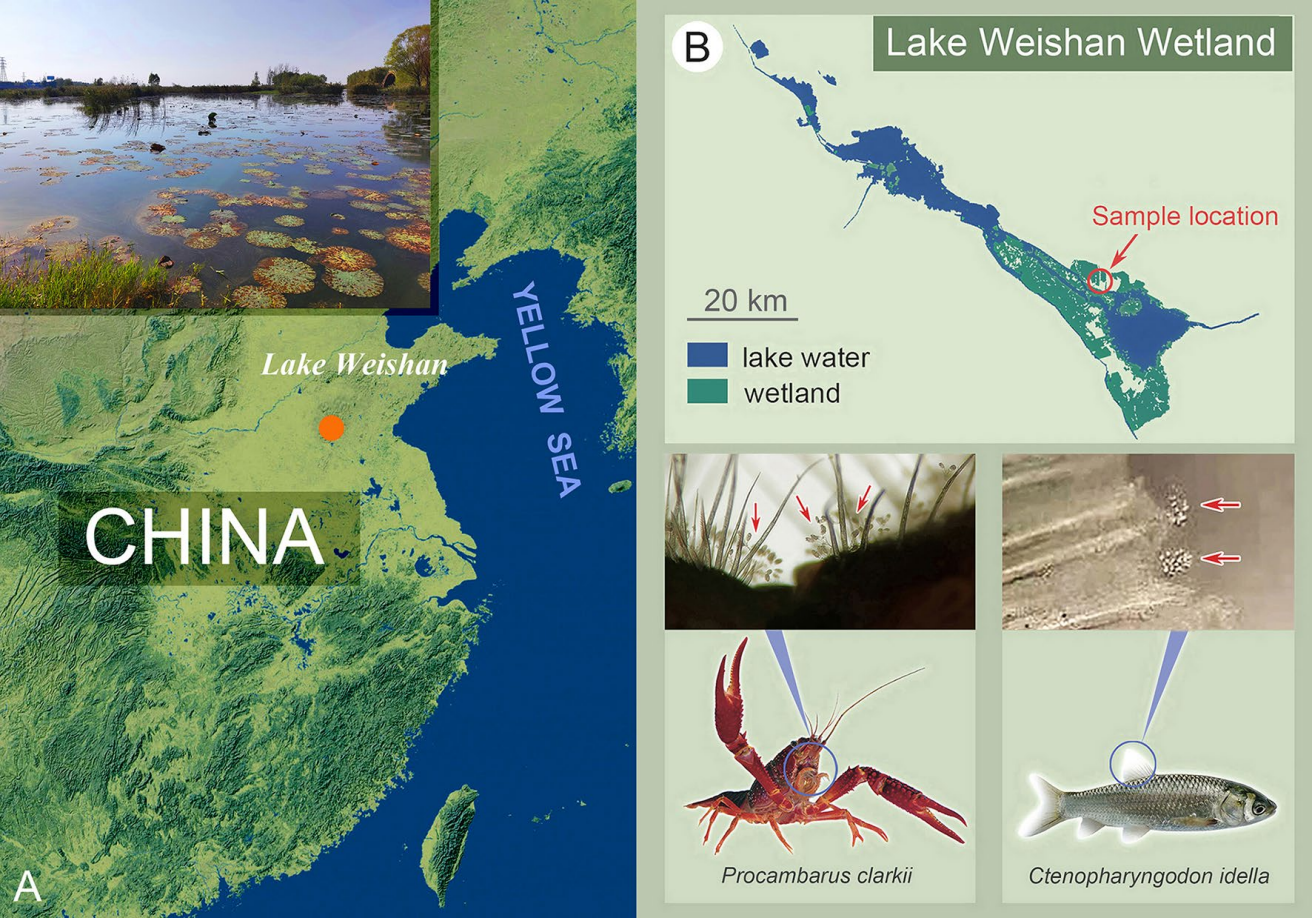
Sampling sites and peritrich-infested hosts. **A** Partial map of China, to indicate the location of the Lake Weishan Wetland. **B** Map of the Lake Weishan Wetland, showing the location of the sampling sites and sites of attachment to hosts

During faunal surveys of freshwater ciliates in Lake Weishan Wetland (Fig. 1), northern China, four species representing three genera, i.e., *Epistylis* Ehrenberg, 1830, *Orborhabdostyla* Foissner et al., 2010 and *Parapiosoma* gen. nov., were investigated using modern methods. Here we provide detailed morphological information of these four species based on observations of specimens in vivo and after silver staining (Table 1). In addition, their phylogenetic relationships inferred from SSU rDNA, ITS1-5.8S-ITS2 and LSU rDNA sequences were analyzed.

**Table 1:** Morphometrical characterization of four species. All data are based on Weishan populations

Character	Species	Max	Min	Mean	SD	CV	n
Zooid length in vivo	*Epistylis cambari*	70	55	59.2	4.17	7.0	12
	*Epistylis lwoffi*	80	55	67.9	8.38	12.3	12
	*Orborhabdostyla gracilis* sp. nov	110	85	99.5	13.83	13.9	10
	*Parapiosoma typicum* gen. nov., sp. nov	90	70	83.9	6.01	7.2	9
Zooid width in vivo	*Epistylis cambari*	40	25	28.8	4.82	17.1	12
	*Epistylis lwoffi*	35	25	30.8	2.89	9.4	12
	*Orborhabdostyla gracilis* sp. nov	25	15	23.5	3.37	14.3	10
	*Parapiosoma typicum* gen. nov., sp. nov	35	30	30.6	1.67	5.5	9
Diameter of peristomial lip in vivo	*Epistylis cambari*	30	25	25.8	1.95	7.6	12
	*Epistylis lwoffi*	40	30	34.2	2.89	8.5	12
	*Orborhabdostyla gracilis* sp. nov	25	20	23.5	2.42	10.3	10
	*Parapiosoma typicum* gen. nov., sp. nov	35	30	32.2	2.64	8.2	9
Height of colony in vivo	*Epistylis cambari*	340	240	295.0	52.60	17.8	4
	*Epistylis lwoffi*	550	300	458.0	100.41	21.9	5
	*Orborhabdostyla gracilis* sp. nov	–	–	–	–	–	–
	*Parapiosoma typicum* gen. nov., sp. nov	–	–	–	–	–	–
No. of silverlines, peristome to trochal band	*Epistylis cambari*	44	36	39.9	2.85	7.1	7
	*Epistylis lwoffi*	53	40	43.0	4.55	10.6	7
	*Orborhabdostyla gracilis* sp. nov	70	59	64.0	5.06	7.9	6
	*Parapiosoma typicum* gen. nov., sp. nov	34	22	29.5	5.20	17.6	4
No. of silverlines, trochal band to scopula	*Epistylis cambari*	16	14	15.2	0.84	5.5	5
	*Epistylis lwoffi*	39	30	35.4	3.26	9.2	7
	*Orborhabdostyla gracilis* sp. nov	25	17	21.2	3.06	14.4	6
	*Parapiosoma typicum* gen. nov., sp. nov	49	44	46.5	3.54	7.6	2

Measurements are in μm

*CV* Coefficient of variation in %, *Max* Maximum, *Min* Minimum, *Mean* Arithmetic mean, *n* Number of specimens investigated, *SD* Standard deviation

## Results

Subclass Peritrichia Stein, 1859

Order Sessilida Kahl, 1933

### Genus *Parapiosoma* gen. nov.

**Diagnosis.** Colonial sessilids with non-contractile stalk; zooids with everted peristomial lip; trochal band conspicuous, located at or above mid-body; macronuleus obconical, located beneath the trochal band; transervse silverlines.

**Type species.*** Parapiosoma typicum* sp. nov.

**Etymology.** Composite of the Greek prefix “para” (derive from) and the generic name *Apiosoma,* referring to the similar zooid shape of these two taxa. Neutral gender.

**Species assigned.** *Parapiosoma typicum* sp. nov., *P. gasterostei* (Fauré-Fremiet, 1905) comb. nov.

### *Parapiosoma typicum* sp. nov. (Figs. 2, 3; Table 2)

**Fig. 2:**
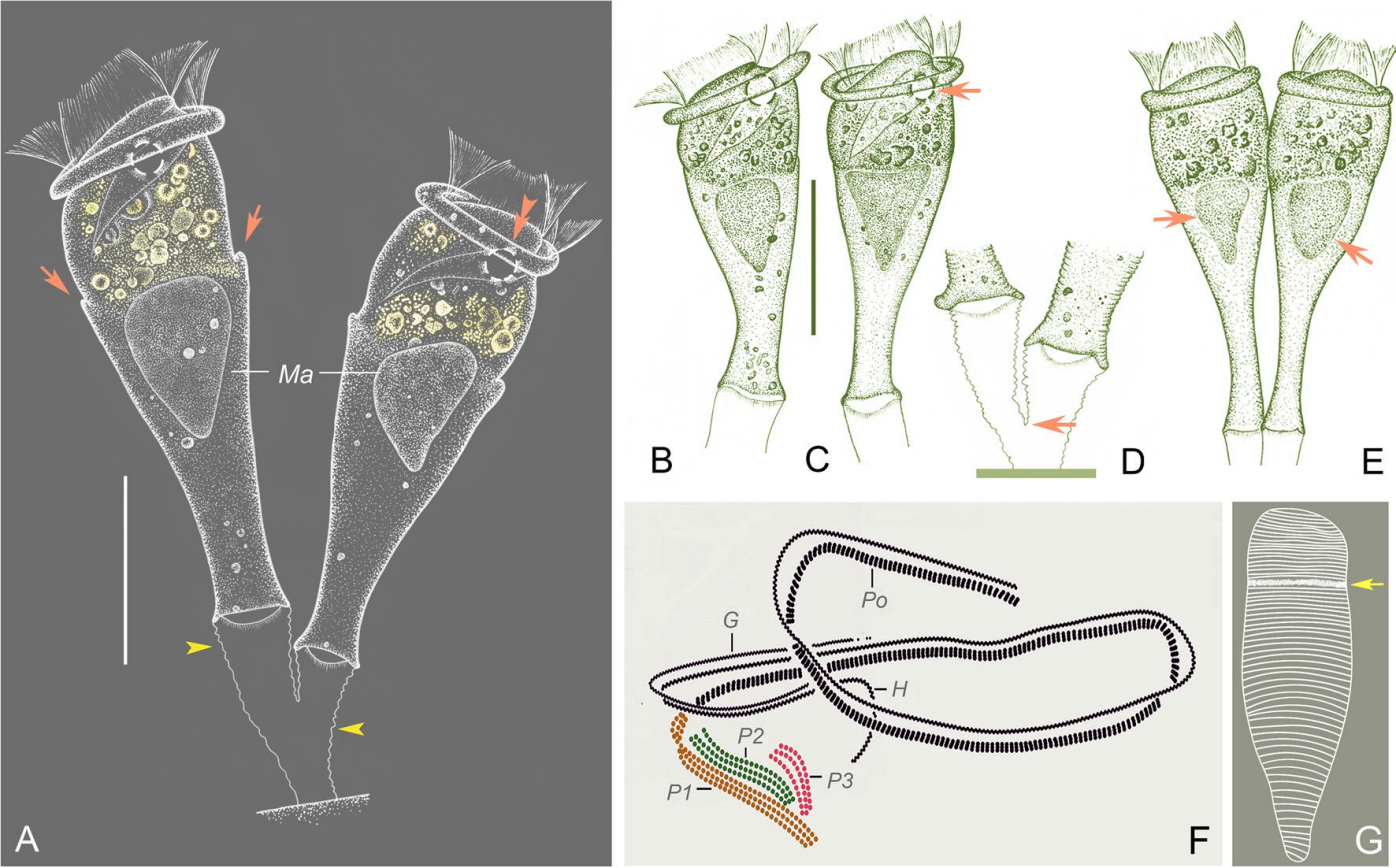
*Parapiosoma typicum* gen. nov., sp. nov. in vivo (**A**–**E**,** G**) and after protargol staining (**F**).** A**,** E** Small colonies, arrows in (**A**) mark the position of the trochal band, arrowheads mark transverse striations of stalk, double arrowheads mark the contractile vacuole, arrows in (**E**) mark the macronucleus.** B**,** C** Solitary zooids, arrow marks the contractile vacuole.** D** Stalk, arrow marks the branching point.** F** Oral ciliature.** G** Pellicular striations, arrow marks the trochal band. *G* Germinal kinety, *H* Haplokinety, *Ma* Macronucleus, *Po* Polykinety, *P1–3* Infundibular polykineties 1–3. Scale bars = 30 μm (**A**), 35 μm (**B**,** C**)

**Fig. 3:**
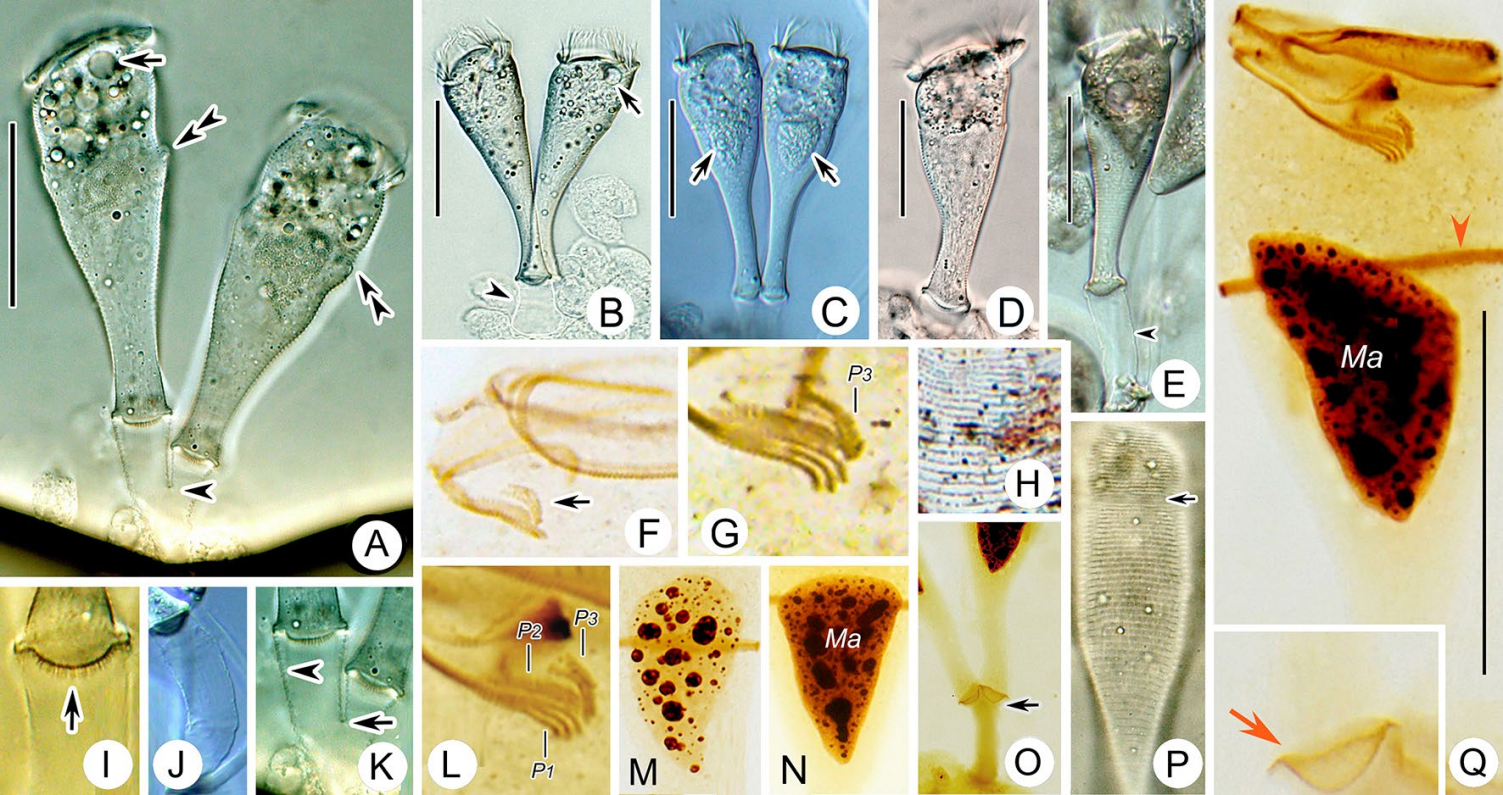
Photomicrographs of *Parapiosoma typicum* gen. nov., sp. nov. in vivo (**A**–**E**,** I**–**K**,** P**), after “dry” silver nitrate staining (**H**) and after protargol staining (**F**,** G**,** L**–**O**,** Q**). **A**–**C** Small colonies, arrows in (**A**,** B**) mark the contractile vacuole, arrows in (**C**) mark the macronucleus, arrowheads mark the stalk, double arrowheads mark the trochal band.** D**,** E** Solitary zooids, arrowhead marks the stalk.** F**,** G** Oral ciliature, arrow marks P3. **H** Part of silverline system.** I**–**K** Stalk, arrow in (**I**) marks the scopula, arrow in (**K**) marks the branching point, arrowhead marks transverse striations.** L** Part of oral ciliature.** M**,** N** Macronucleus.** O** Part of protargol-stained zooids, arrow marks the scopula.** P** Pellicular striations, arrow marks the trochal band.** Q** Holotype specimen, showing the ciliature, arrow marks the scopula, arrowhead marks the trochal band. *Ma* Macronucleus, *P1–3* Infundibular polykineties 1–3. Scale bars = 40 μm (**A**−**E**), 35 μm (**Q**)

**Table 2:** Comparison of *Parapiosoma typicum* gen. nov., sp. nov. with its only congener

Species	Zooid length in vivo	Zooid width in vivo	Stalk surface smooth	Data source
*P. typicum* gen. nov., sp. nov	70–90	30–35	No	Present study
*P. gasterostei*	40–70	22–34	Yes	Scheubel (1973)

Measurements are in μm

**Diagnosis.** Zooids goblet-shaped, about 70–90 × 30–35 μm in vivo. Stalk thick and transparent, with conspicuous transverse striations. Contractile vacuole ventrally located at same level as, or slightly beneath, peristomial lip. Macronucleus conical, located in mid-region of zooid. Infundibular polykinety 3 consists of three equal-length rows of kinetosomes, terminates adstomally above infundibular polykinety 1. Silverlines numbering about 22–34 above and 44–49 below trochal band. Freshwater habitat.

**Etymology.** The species-group name “typicum” refers to the type species of *Parapiosoma*.

**Type locality.** Freshwater aquaculture pond in Lake Weishan (N34°44′21.44″; E117°09′33.80″), Shandong Province, China.

**Host species and site of attachment.** Grass carp *Ctenopharyngodon idella* (Cypriniformes, Cyprinidae). *Parapiosoma typicum* sp. nov. was found attached to the fins.

**Type material.** One holotype slide (number: WT2020121601–01) with protargol-stained specimens and one paratype slide (number: WT2020121601–02) with “dry” silver nitrate specimens were deposited in the Laboratory of Marine Protozoan Biodiversity and Evolution, Shandong University, Weihai, China. One paratype slide (number: WT2020121601–03) with protargol-stained specimens was deposited in the Laboratory of Protozoology, Ocean University of China (OUC), Qingdao, China.

**Description.** Zooids goblet-shaped, about 70–90 × 30–35 μm in vivo (Figs. 2A–C, E, 3A–E). Peristomial lip single-layered, about 30–35 μm in width (Figs. 2A, C, E, 3A–E). Peristomial disc is obliquely elevated above peristome in fully extended zooids (Figs. 2A–C, 3A, D). Stalks are transparent, sometimes dichotomously branched (Figs. 2A, D, 3A, E, K, O). Primary stalk is about 15 μm in width, with conspicuous transverse striations (Figs. 2A, D, 3A, B, E, I–K). Pellicular striations fine (Figs. 2G, 3P).

Cytoplasm colourless, usually with numerous vacuoles containing yellow or grey contents. Contractile vacuole located at same level as, or slightly beneath, peristomial lip and near ventral wall of infundibulum, which is about 12 μm in diameter (Figs. 2A–C, 3A, B). Macronucleus obconical, located in mid-region of zooid (Figs. 2A–C, E, 3C, M, N, Q).

Haplokinety and polykinety perform about 1.25 circuits around peristome and a further turn within infundibulum (Figs. 2F, 3F, Q). Each of the three infundibular polykineties (P1–3) consists of three rows of kinetosomes (Figs. 2F, 3F, G, L, Q). Three rows of P1 nearly equal in length (Figs. 2F, 3G, L, Q). P2 adstomally terminates at convergence of P1 and P3 (Figs. 2F, 3F, G, L, Q) and abstomally separates from P1, with its outer row separated abstomally from the other two rows (Figs. 2F, 3G, L). P3 terminates adstomally above P1, which is composed of three equal-length rows of kinetosomes (Figs. 2F, 3F, G, L, Q). Epistomial membrane was not observed (Figs. 2F, 3Q). Germinal kinety and haplokinety are parallel in upper two-thirds of infundibulum (Figs. 2F, 3G, Q). Trochal band consists of a row of dikinetids, located about 40% down length of zooid (Fig. 3P, Q). Silverline system consists of closely spaced transverse silverlines, numbering 22–34 (n = 4) above and about 44–49 (n = 2) below trochal band (Figs. 2G, 3H, P).

Family: Epistylididae Kahl, 1933

Genus: *Orborhabdostyla* Foissner et al., 2010

### *Orborhabdostyla gracilis* sp. nov. (Figs. 4, 5; Table 3)

**Diagnosis.** Zooids slender, about 85–110 × 15–25 μm in vivo. Stalk thin, with conspicuous longitudinal striations. Peristomial disc clearly elevated above peristome. Contractile vacuole dorsally located beneath peristomial lip. Macronucleus transversely oriented. Infundibular polykinety 3 consists of three equal-length rows of kinetosomes, terminates adstomally above infundibular polykinety 1. Silverlines numbering about 59–70 from peristome to trochal band and about 17–25 from trochal band to scopula. Freshwater habitat.

**Fig. 4:**
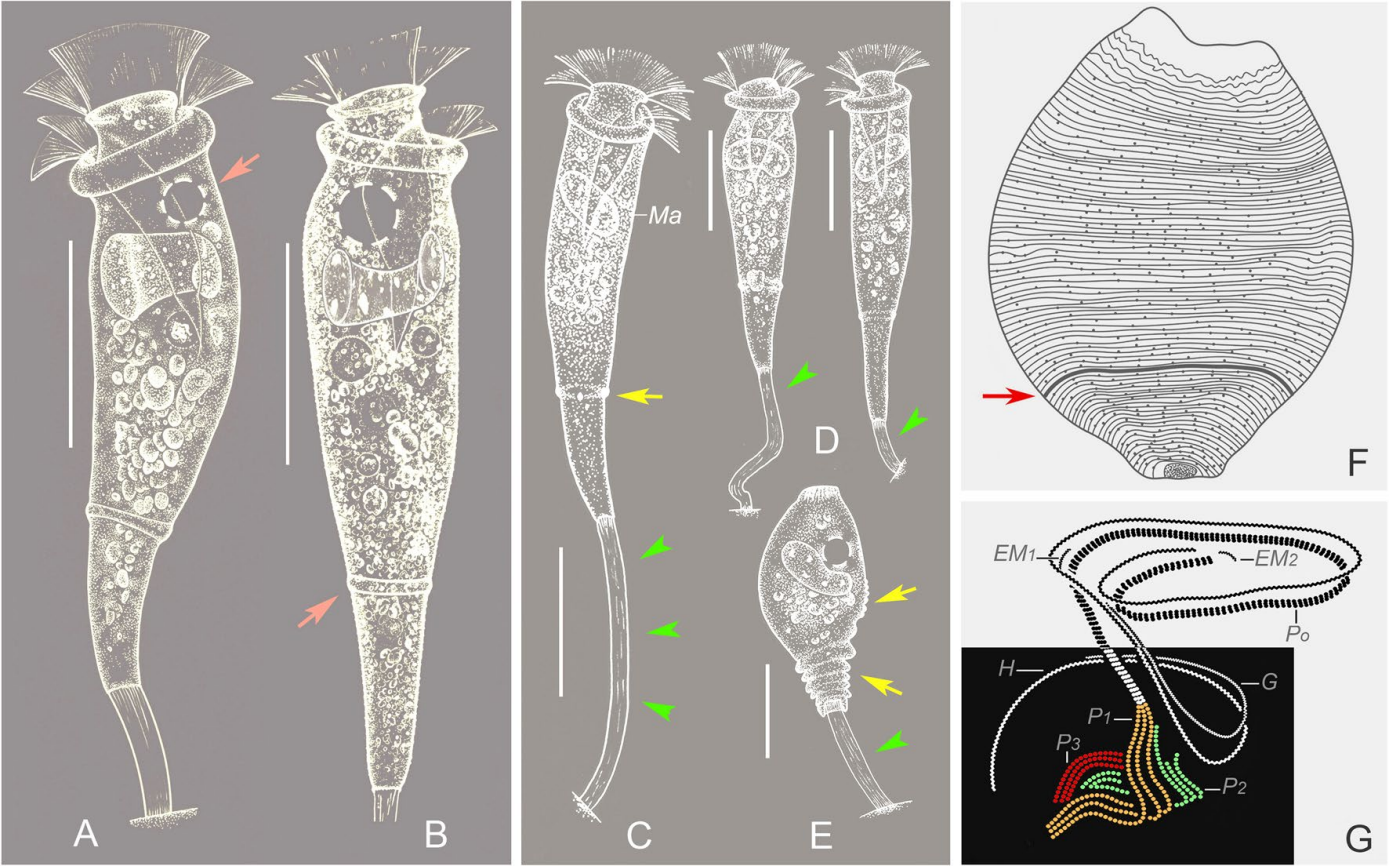
*Orborhabdostyla gracilis* sp. nov. in vivo (**A**–**E**), after “dry” silver nitrite staining (**F**) and after protargol staining (**G**). **A**–**D** Zooids, arrow in (**A**) marks the contractile vacuole, arrows in (**B**, **C**) mark the trochal band, arrowheads mark the stalk. **E** Contracted zooid, arrows mark folds, arrowhead marks the stalk. **F** Silverline system, arrow marks the trochal band. **G** Oral ciliature. *EM1, 2* Epistomial membranes 1, 2, *G* Germinal kinety, *H* Haplokinety, *Ma* Macronucleus, *Po* Polykinety, *P1–3* Infundibular polykineties 1–3. Scale bars = 40 μm (**A**,** B**), 35 μm (**C**, **D**), 25 μm (**E**)

**Fig. 5:**
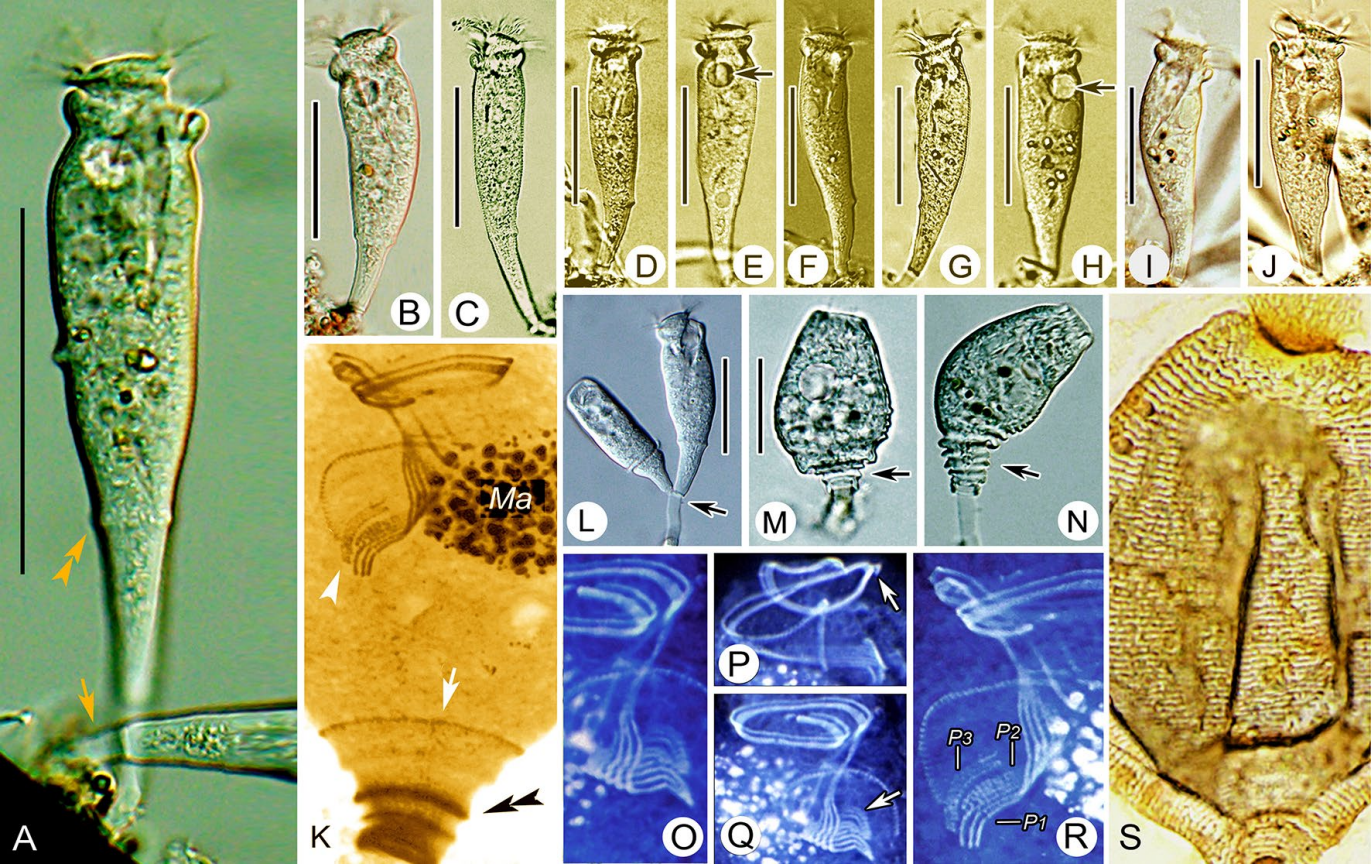
Photomicrographs of *Orborhabdostyla gracilis* sp. nov. in vivo (**A**–**J**, **L**–**N**), after protargol staining (**K**, **O**–**R**) and after “dry” silver nitrate staining (**S**). **A**–**J** Different zooids, showing the variation of shape, arrow in (**A**) marks the stalk, double arrowheads in (**A**) mark the trochal band, arrows in (**E**, **H**) mark the contractile vacuole. **K** Holotype specimen, showing the ciliature, arrowhead marks P3, arrow marks the trochal band, double arrowheads mark folds. **L** Zooids at the end of binary fission, arrow marks the stalk. **M**, **N** Contracted zooids, arrows mark folds. **O** Protargol-stained zooid (processed by the reverse function via Photoshop), showing the macronucleus. **P**–**R** Oral ciliature (processed by the reverse function via Photoshop), arrow in (**P**) marks the epistomial membrane 1, arrow in (**Q**) marks P3. **S** Silverline system. Abbreviations: *Ma*, Macronucleus, *P1–3*, Infundibular polykineties 1–3. Scale bars = 50 μm (**A**–**J**), 35 μm (**L**), 30 μm (**M**)

**Table 3:** Comparison of *Orborhabdostyla gracilis* sp. nov. with similar congeners

Species	Zooid length in vivo	Zooid width in vivo	Zooid shape	CV	Shape of contracted zooid	Data source
*O. gracilis* sp. nov.	85–110	15–25	Conical	Dorsal	Oval	Present study
*O. bromelicola*	50–75	13–18	Conical	Dorsal	Oval	Foissner et al. (2010)
*O. kahli*	60–70	–	Cylindrical	Ventral	–	Kahl (1935)
*O. brevipes*	80–90	–	–	–	Cylindrical	Claparѐde and Lachmann (1858)

Measurements are in μm

*CV* Contractile vacuole, *–* Data not available

**Etymology.** The species-group name “*gracilis*” means “slender” and refers to the slender zooid shape of this species.

**Type locality.** Lake Weishan (N34°45′8.63″; E117°09′0.51″), Shandong Province, China.

**Type material.** One holotype slide (number: WT2021061801–01) with protargol-stained specimens and one paratype slide (number: WT2021061801–02) with “dry” silver nitrate specimens were deposited in the Laboratory of Marine Protozoan Biodiversity and Evolution, Shandong University, Weihai, China. One paratype slide (number: WT2021061801–03) with protargol-stained specimens was deposited in the Laboratory of Protozoology, OUC, Qingdao, China.

**Description.** Zooids slender, about 85–110 × 15–25 μm in vivo (Figs. 4A–D, 5A–J). Peristomial lip single-layered, about 20–25 μm in width (Figs. 4A–D, 5A–J). Peristomial disc is obliquely elevated above peristome in fully extended zooids (Figs. 4A–D, 5A–J). Contracted zooids ellipsoidal, with transverse pellicular folds in posterior half (Figs. 4E, 5M, N). Primary stalk about 5 μm across, with conspicuous longitudinal striations (Figs. 4A, B, 5L). Pellicular striations transverse and extremely fine (Fig. 5S).

Cytoplasm colourless, often with numerous vacuoles containing with yellow and/or green contents. Contractile vacuole about 12 μm in diameter, located beneath peristomial lip and near dorsal wall of infundibulum (Figs. 4A, B, 5E, H). Macronucleus curved, with one margin frequently folded inwards, transversely oriented (Figs. 4A–E, 5O).

Haplokinety and polykinety perform about 1.25 circuits around peristome and a further turn within infundibulum (Figs. 4G, 5K, P–R). Each of the three infundibular polykineties (P1–3) consists of three rows of kinetosomes (Figs. 4G, 5K, P–R). Three rows of P1 are nearly equal in length (Figs. 4G, 5Q, R). P2 terminates adstomally at convergence of P1 and P3 (Figs. 4G, 5K, Q, R). Inner row of P2 abstomally converges with P1, mid-row of P2 abstomally converges with inner row (Figs. 4G, 5Q). Outer row of P2 abstomally separated from the other two rows (Figs. 4G, 5Q). P3 terminates adstomally above P1, which is composed of three equal-length rows (Figs. 4G, 5K, Q, R). Two epistomial membranes (EM 1, 2): EM1 near entrance of infundibulum; EM 2 near distal ends of haplokinety and polykinety (Figs. 4G, 5P, Q). Germinal kinety and haplokinety are parallel in upper two-thirds of infundibulum (Figs. 4G, 5R). Trochal band comprises a dikinetidal row, located about 75% down length of zooid (Figs. 4F, 5K). Silverline system consists of closely spaced transverse silverlines, numbering about 59–70 (n = 6) above trochal band and about 17–25 (n = 6) below trochal band (Figs. 4F, 5S).

Genus: *Epistylis* Ehrenberg, 1830.

### *Epistylis cambari* Kellicott, 1885 (Figs. 6, 7; Table 4)

*Epistylis cambari* has been redescribed several times since it was originally discovered by Kellicott (1885). However, its superficial description, lack of information on its ciliature and lack of gene sequence data necessitates a reinvestigation using modern techniques. Here, we provide a detailed redescription and an improved diagnosis.

**Fig. 6:**
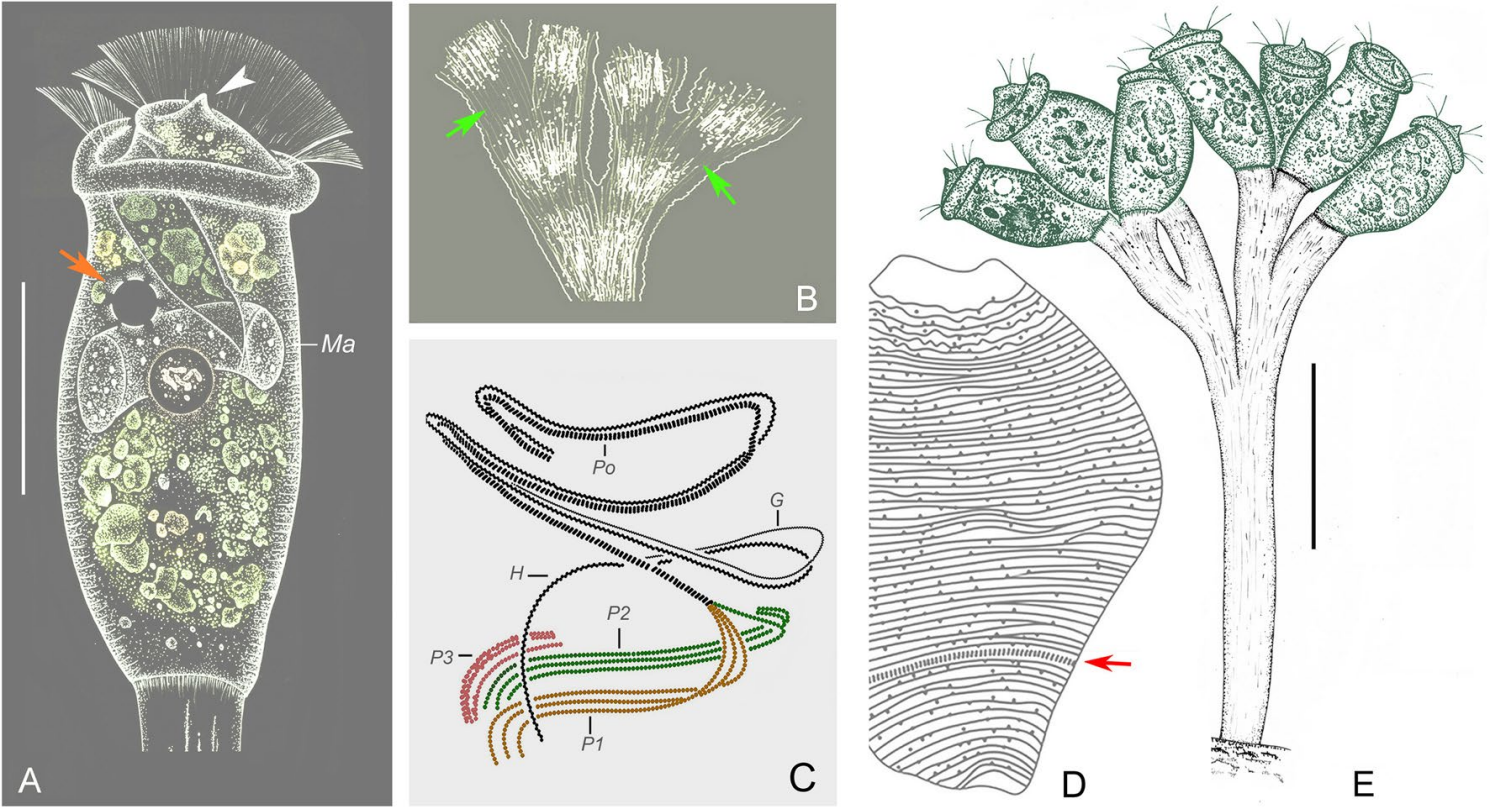
*Epistylis cambari *in vivo (**A**, **B**, **E**), after protargol staining (**C**) after and “dry” silver nitrite staining (**D**). **A** Zooid, arrow marks the contractile vacuole, arrowhead marks the conical protuberance on peristomial disc. **B** Part of stalk, arrows mark the longitudinal striations. **C** Oral ciliature. **D** Silverline system, arrow marks the trochal band. **E** A mature colony. *G* Germinal kinety, *H* Haplokinety, *Ma* Macronucleus, *Po* Polykinety, *P1–3* Infundibular polykineties 1–3. Scale bars = 30 μm (**A**), 80 μm (**E**)

**Fig. 7:**
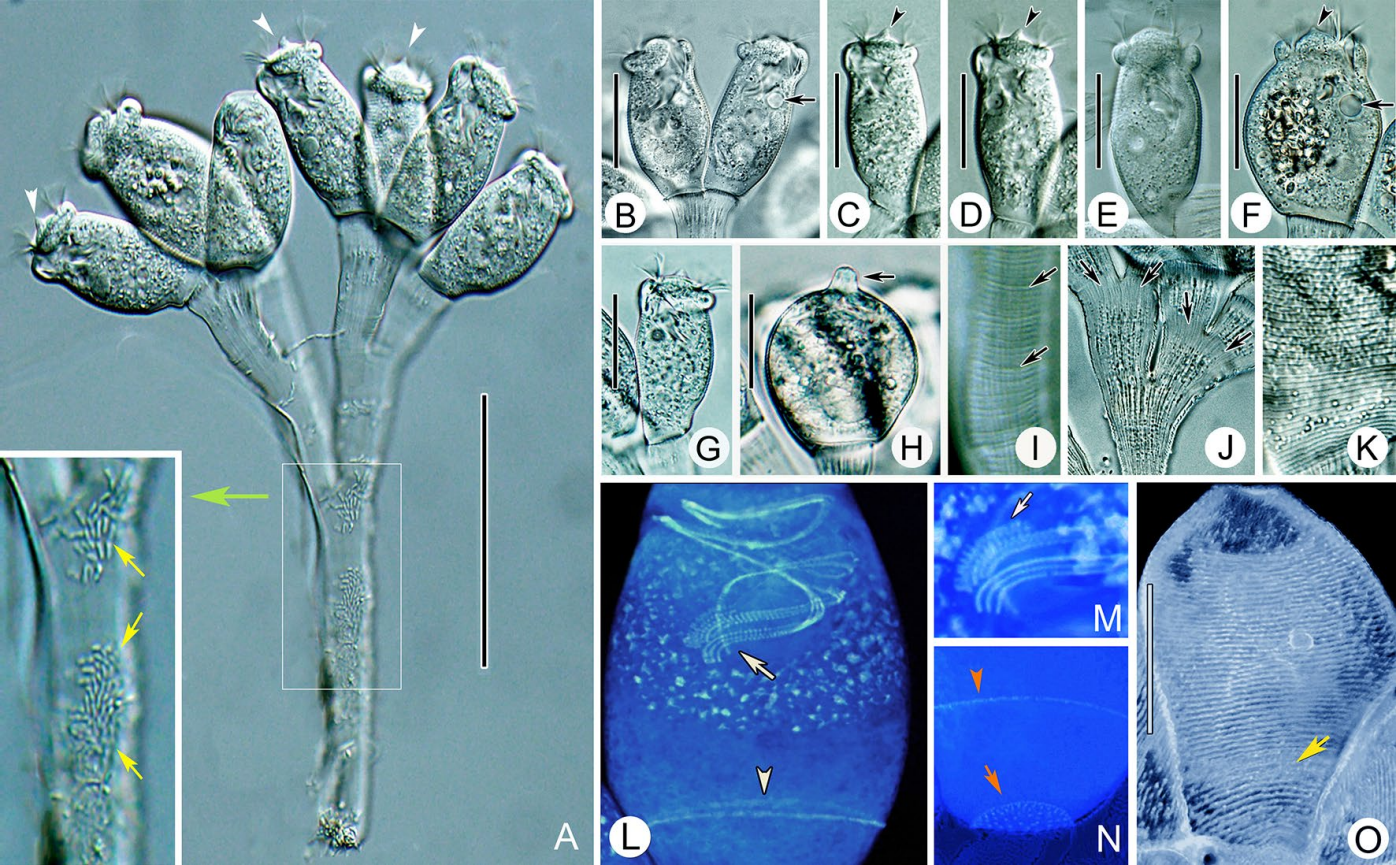
Photomicrographs of *Epistylis cambari *in vivo (**A**–**K**), after protargol staining (**L**–**N**) and after “dry” silver nitrate staining (**O**). **A** Mature colony, arrows mark the rod-like bacteria in surface of stalk, arrowheads mark the conical protuberance on the peristomial disc. **B**–**G** Different zooids, showing the variation of shape, arrows mark the contractile vacuole, arrowheads mark the conical protuberance on peristomial disc. **H** Contracted zooid, arrow marks the snout-like projection. **I**, **J** Stalk, arrows in (**I**) mark the transverse striations, arrows in (**J**) mark the longitudinal striations. **K** Pellicular striations. **L** Protargol-stained zooids (processed by the reverse function via Photoshop), showing the ciliature, arrow marks P1, arrowheads mark the trochal band. **M** Part of oral ciliature, arrow marks P3. **N** Part of protargol-stained zooid, arrow marks the scopula, arrowhead marks the trochal band. **O** Silverline system (processed by the reverse function via Photoshop), arrow marks the trochal band. Scale bars = 80 μm (**A**), 30 μm (**B**–**G**), 20 μm (**H**), 25 μm (**O**)

**Table 4:** Comparison of *Epistylis cambari* (Weishan population) with similar congeners and another population

Species	Zooid length in vivo	Zooid width in vivo	PD with a protuberance	CV	Stalk with annular swellings	Data source
*E. cambari*	55–70	25–40	Yes	Ventral	No	Present study
*E. cambari*	50	–	Yes	Ventral	No	Kellicott (1885)
*E. crassicolis*	60–100	27–44	Yes^a^	Ventral^a^	Yes	Schödel (2018)
*E. microdiscum*	45–55	30–35	No	Dorsal	No	Stiller (1963)
*E. sommerae*	48–57	29–38	Yes	Ventral	No	Sommer (1951); Schödel (1987)
*E. vasta*	50–104	38–60	No	Ventral	No	Sommer (1951)

Measurements are in μm

*CV* Contractile vacuole, *PD* Peristomial disc, *–* Data not available

^a^Contradictory in different reports, need to be reconfirmed

**Improved diagnosis.** Colony up to 340 μm high. Stalk symmetrically dichotomously branched, stout, with outer transverse and inner longitudinal striations. Zooids cylindrical, about 55–70 × 25–40 μm in vivo. Peristomial disc moderately elevated above peristome, with a conical protuberance in its centre. Contractile vacuole located ventrally, beneath peristomial lip. Macronucleus C-shaped, usually transversely oriented. Infundibular polykinety 3 consists of three equal-length rows of kinetosomes, terminates adstomally above infundibular polykinety 1. Silverlines numbering about 36–44 above and about 14–16 below trochal band. Freshwater habitat.

**Voucher slides.** Two protargol slides (numbers: WT2021061102–01, WT2021061102–02), and one “dry” silver nitrate slide (number: WT2021061102–03) were deposited in the Laboratory of Protozoology, OUC, Qingdao, China.

**Description based on Weishan population.** Colony up to 340 μm high, usually with fewer than 10 zooids (Figs. 6E, 7A). Stalk symmetrically dichotomously branched, with transverse striations on surface and discontinuous rough longitudinal striations in the fibrillar matrix (Figs. 6B, 7A, I, J). Primary stalk about 15 μm across and often incrassate beneath branching points (Figs. 6B, 7J).

Zooids cylindrical, usually 55–70 × 25–40 μm in vivo (Figs. 6A, 7B–G). Peristomial lip single-layered, about 25–30 μm across (Figs. 6A, 7B–G). Peristomial disc moderately elevated above peristome, which has a conical protuberance in its centre (Figs. 6A, 7C, D, F, G). Contracted zooids ovoidal, with a snout-like projection at anterior end; posterior half without transverse folds (Fig. 7H). Pellicular striations conspicuous when were viewed at magnifications of 400 × or higher (Fig. 7K).

Cytoplasm colourless, usually containing small grey or transparent food granules. Contractile vacuole about 7 μm in diameter, located beneath peristomial lip and near ventral wall of infundibulum (Figs. 6A, 7B, F). Macronucleus C-shaped, usually transversely oriented (Figs. 6A, 7L).

Haplokinety and polykinety perform about 1.25 circuits around peristome and a further turn within infundibulum (Figs. 6C, 7L). Each infundibular polykinety (P1–3) composed of three rows of kinetosomes (Figs. 6C, 7L, M). Three rows of P1 equal-length (Figs. 6C, 7L, M). P2 terminates adstomally at convergence of P1 and P3, its inner row abstomally converging with P1 (Figs. 6C, 7L, M). Three rows of P3 nearly equal-length, terminates slightly above P1 (Figs. 6C, 7L, M). Epistomial membrane not observed. Germinal kinety and haplokinety lie in parallel in upper two-thirds of infundibulum (Figs. 6C, 7L). Trochal band consists of a row of dikinetids, located about 80% down length of zooid (Figs. 6D, 7O). Silverline system consists of transverse silverlines numbering about 36–44 (n = 7) between peristome and trochal band, and about 14–16 (n = 5) between trochal band and scopula (Figs. 6D, 7O).

### ***Epistylis lwoffi*** Fauré-Fremiet, 1943 (Figs. 8, 9; Table 5)

*Epistylis lwoffi* is a common epibiotic species that was first reported by Fauré-Fremiet (1943). It has been subsequently isolated from the surface of various freshwater fishes, but details of its morphology are still incomplete and gene sequence data are lacking. Here, we provide a detailed redescription and an improved diagnosis of this species.

**Fig. 8:**
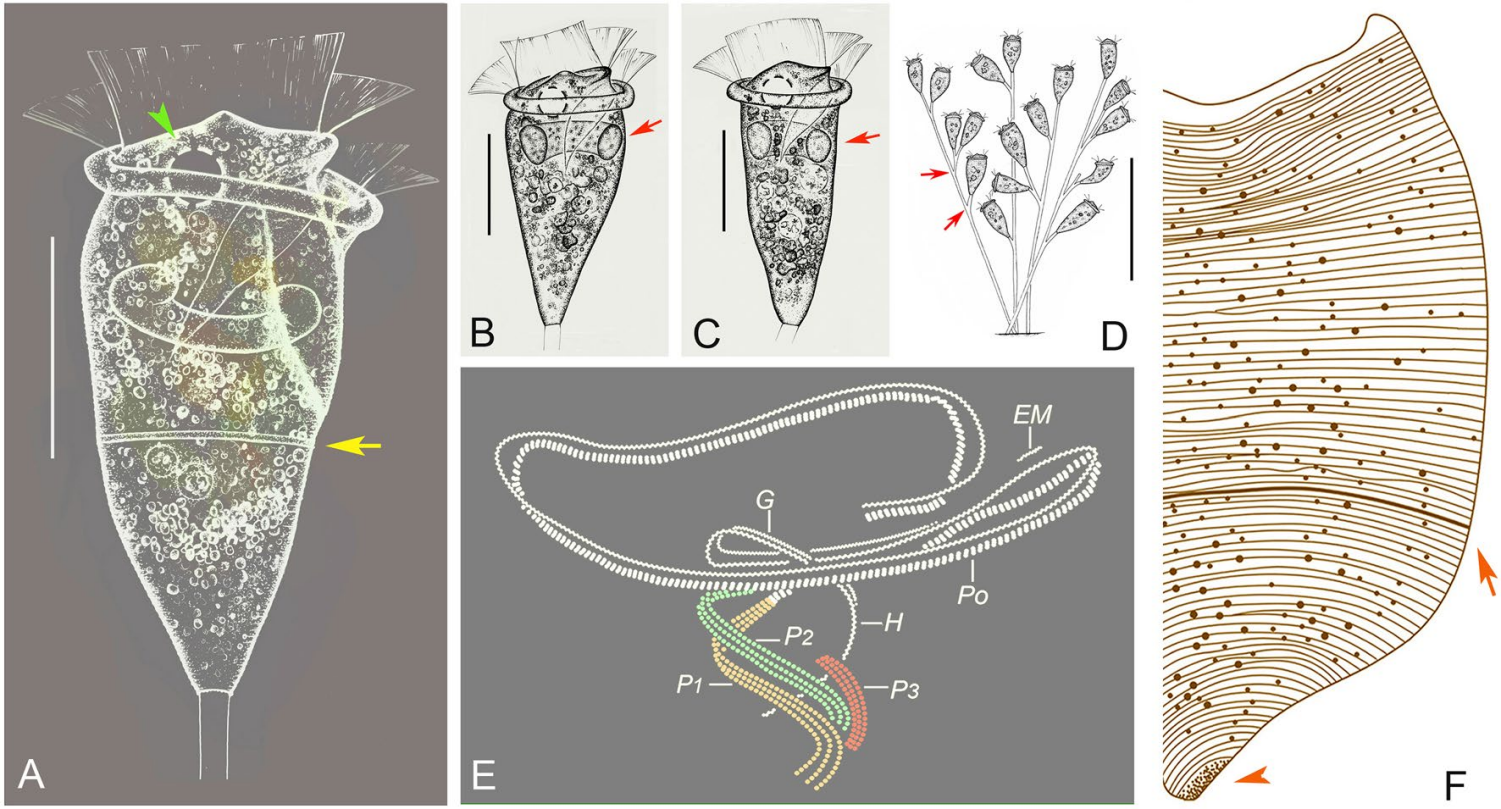
*Epistylis lwoffi *in vivo (**A**–**D**), after protargol staining (**E**) and after “dry” silver nitrite staining (**F**). **A**–**C** Zooids, arrow in (**A**) marks the trochal band, arrows in (**B**, **C**) mark the macronucleus, arrowhead marks the contractile vacuole. **D** Mature colonies, arrows mark the branching points. **E** Oral ciliature. **F** Silverline system, arrow marks the trochal band, arrowhead marks the scopula. *EM* Epistomial membrane, *G* Germinal kinety, *H* Haplokinety, *Po* Polykinety, *P1–3* Infundibular polykineties 1–3. Scale bars = 30 μm (**A**–**C**), 150 μm (**D**)

**Fig. 9:**
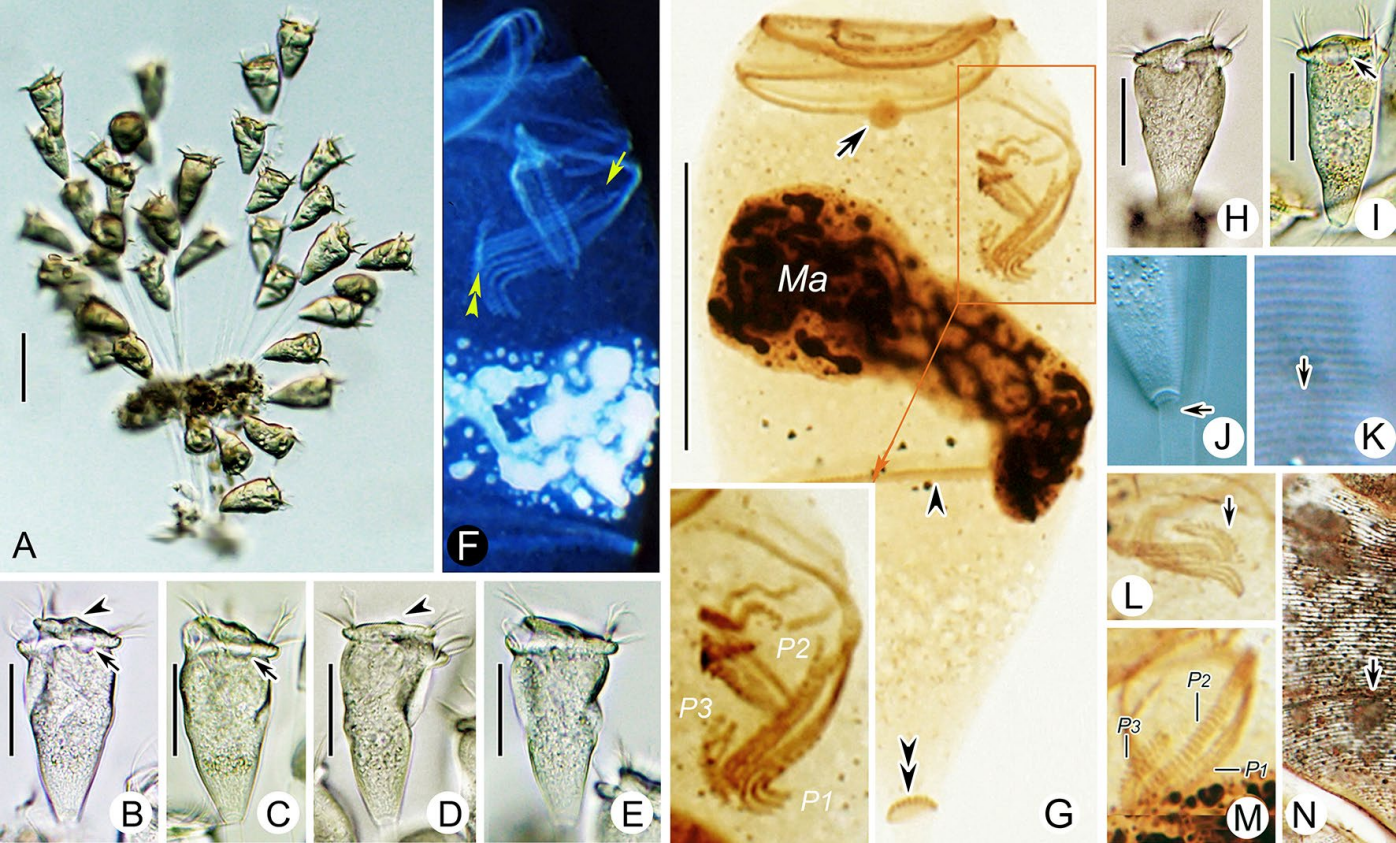
Photomicrographs of *Epistylis lwoffi *in vivo (**A**–**E**, **H**–**K**), after protargol staining (**F**, **G**, **L**, **M**) and after “dry” silver nitrate staining (**N**). **A** Mature colonies. **B**–**E**, **H**, **I** Different zooids, showing the variation of shape, arrows mark the contractile vacuole, arrowheads mark the conical protuberance in peristomial disc. **F** Oral ciliature (processed by the reverse function via Photoshop), arrow marks abstomal end of P2, double arrowheads mark P3. **G** Protargol-stained zooid, showing the ciliature, arrow marks the micronucleus, arrowhead marks the trochal band, double arrowheads mark the scopula. **J** Stalk, arrow marks the branching points. **K** Pellicular striations, arrow marks the trochal band. **L**, **M** Part of oral ciliature, arrow marks P3. **N** Silverline system, arrow marks the trochal band. *Ma* Macronucleus, *P1–3* Infundibular polykineties 1–3. Scale bars = 70 μm (**A**), 35 μm (**B**–**E**, **H**, **I**), 25 μm (**G**)

**Table 5:** Comparison of *Epistylis lowffi* (Weishan population) with similar congeners and other populations

Species	Zooid length in vivo	Zooid width in vivo	CV	Ma	Regularly dichotomously branched stalk	No. of silverlines^a^	No. of rows of P3	Data source
*E. lwoffi*	55–80	35–35	Dorsal	Transverse	No	70–82	3	Present study
*E. lwoffi*	65–75	35	Dorsal	Transverse	No	–	–	Fauré-Fremiet (1943)
*E. lwoffi*	50	20	Ventral	Transverse	–	–	–	Lom and Vávra (1961)
*E. lwoffi*	40–80	25–45	Dorsal	Transverse	–	–	–	Scheubel (1973)
*E. lwoffi*	49–70	31–49	Dorsal	Transverse	No	81–96	2	Foissner (1983)
*E. anastatica*	60–100	20–35	Ventral	Longitudinal	Yes	56–87	2	Lu et al. (2020)
*E. plicatilis*	90–160	25–50	Dorsal	Transverse	Yes	176–206	3^b^	Foissner et al. (1992)

Measurements are in μm

*CV* Contractile vacuole, *Ma* Macronucleus, *P3* Infundibular polykinety 3, – Data not available

^a^From peristome to scopula

^b^Contradictory in different reports, need to be reconfirmed

**Improved diagnosis.** Colony up to 550 μm high. Stalk dichotomously branched, smooth and thin. Zooids usually inverted bell-shaped, about 40–80 × 20–49 μm in vivo. Peristomial disc clearly elevated above peristome, usually with a wart-like protuberance in its centre. Contractile vacuole dorsally located at same level as peristomial lip. Macronucleus C-shaped, transversely oriented. Infundibular polykinety 3 consists of three equal-length rows of kinetosomes, terminates adstomally above infundibular polykinety 1. Silverlines numbering about 40–53 from peristome to trochal band and about 30–39 from trochal band to scopula.

**Voucher slides.** Two protargol slides (numbers: WT2020121602–01, WT2020121602–02) and one “dry” silver nitrate slide (number: WT2020121602–03) containing voucher specimens were deposited in the Laboratory of Protozoology, OUC, Qingdao, China.

**Description based on Weishan population.** Colony up to 550 μm high, usually containing 8–16 zooids (Figs. 8D, 9A). Stalk dichotomously branched, basal portion about 5 μm in width (Figs. 8D, 9A, J). Accessory stalks short, about 5 μm in width (Figs. 8D, 9A). Zooids usually located on only one side of accessory stalk (Figs. 8D, 9A, J).

Zooids usually inverted bell-shaped, about 55–80 × 25–40 μm in vivo (Figs. 8A–C, 9B–E, H, I). Peristomial lip single-layered, about 30–40 μm across (Figs. 8A–C, 9B–E, H, I). Peristomial disc elevated obliquely above peristome in fully extended zooids, with a wart-like protuberance in its centre (Figs. 8A–C, 9B, D). Pellicular striations extremely fine (Fig. 9K).

Cytoplasm colourless, usually containing numerous vacuoles with yellow and/or green contents. Contractile vacuole about 9 μm in diameter, located at same level as peristomial lip and near dorsal wall of infundibulum (Figs. 8A–C, 9B, C, I). Macronucleus C-shaped, transversely oriented (Figs. 8A–C, 9G). Single micronucleus (Fig. 9G).

Haplokinety and polykinety perform about 1.25 circuits around peristome and a further turn within infundibulum (Figs. 8E, 9G). Each of the three infundibular polykineties (P1–3) composed of three rows of kinetosomes (Figs. 8E, 9F, G, L, M). Three rows of P1 nearly equal length (Figs. 8E, 9F, G, M). P2 terminates adstomally at convergence of P1 and P3 (Figs. 8E, 9F, G, M). Inner row of P2 abstomally converges with P1, mid-row of P2 abstomally converges with inner row (Figs. 8E, 9F, G). Outer row of P2 separates abstomally from the other two rows (Figs. 8E, 9F, G). Three rows of P3 equal-length, terminates adstomally above P1 (Figs. 8E, 9G, L). One epistomial membrane, located near entrance of infundibulum (Figs. 8E, 9G). Germinal kinety and haplokinety lie in parallel in upper two-thirds of infundibulum (Figs. 8E, 9G). Trochal band consists of a row of dikinetids, located about 60% down length of zooid (Figs. 8F, 9G). Silverline system consists of closely spaced transverse silverlines numbering about 40–53 (n = 7) above trochal band and about 30–39 (n = 7) below trochal band (Figs. 8F, 9N).

### Molecular data and phylogenetic analyses (Figs. 10, 11, 12, 13)

**Fig. 10:**
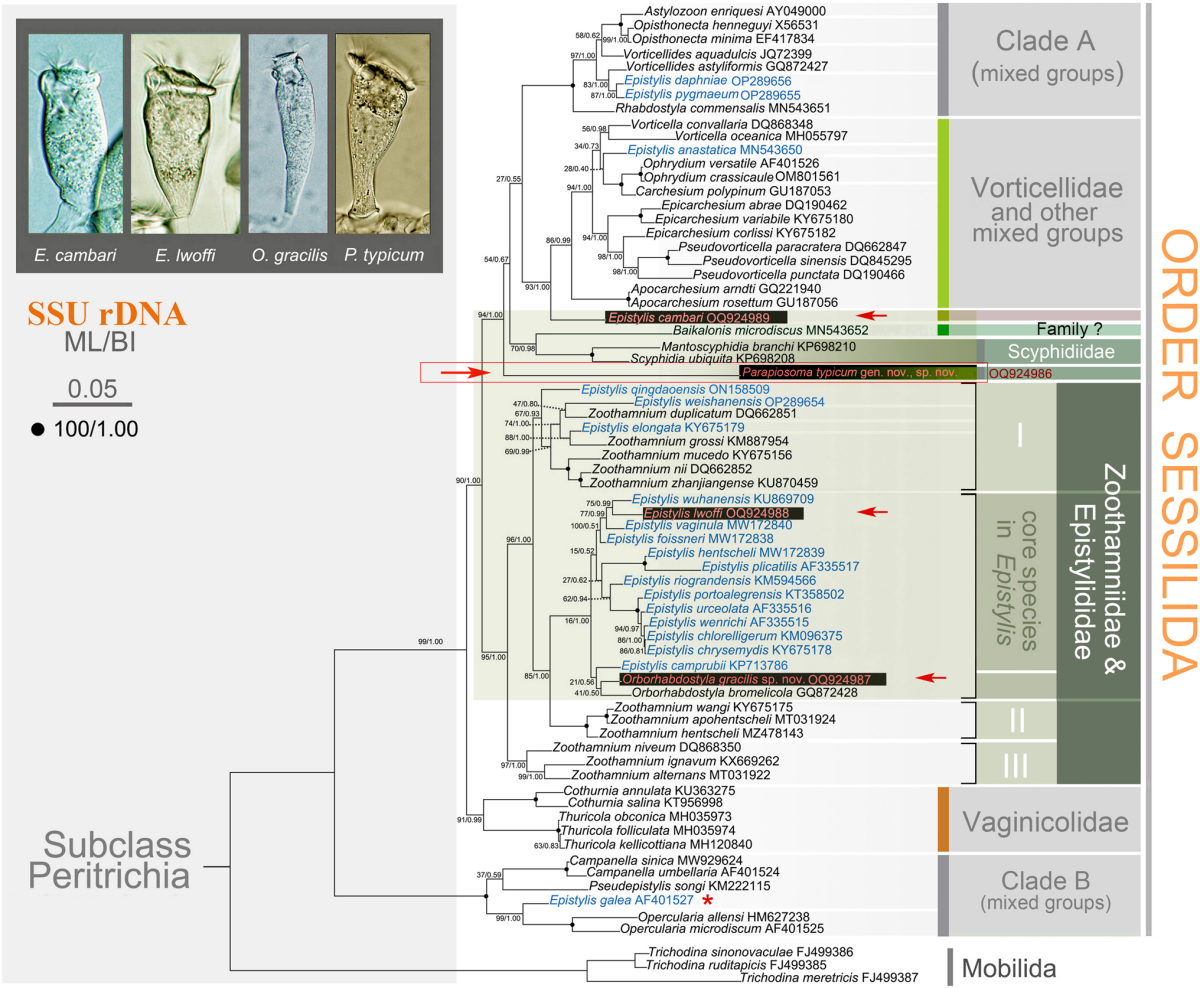
ML tree based on SSU rDNA sequences, revealing the phylogenetic positions of *Epistylis cambari*, *E*. *lwoffi*, *Orborhabdostyla gracilis* sp. nov., and *Parapiosoma typicum* gen. nov., sp. nov. (arrows). Numbers near nodes denote maximum bootstrap values of ML out of 1000 replicates and posterior probabilities of Bayesian inference (BI). Sequence of “*Epistylis galea*” (AF401527, marked with asterisk) might be misidentified. The scale bar indicates five substitutions per 100 nucleotide positions

**Fig. 11:**
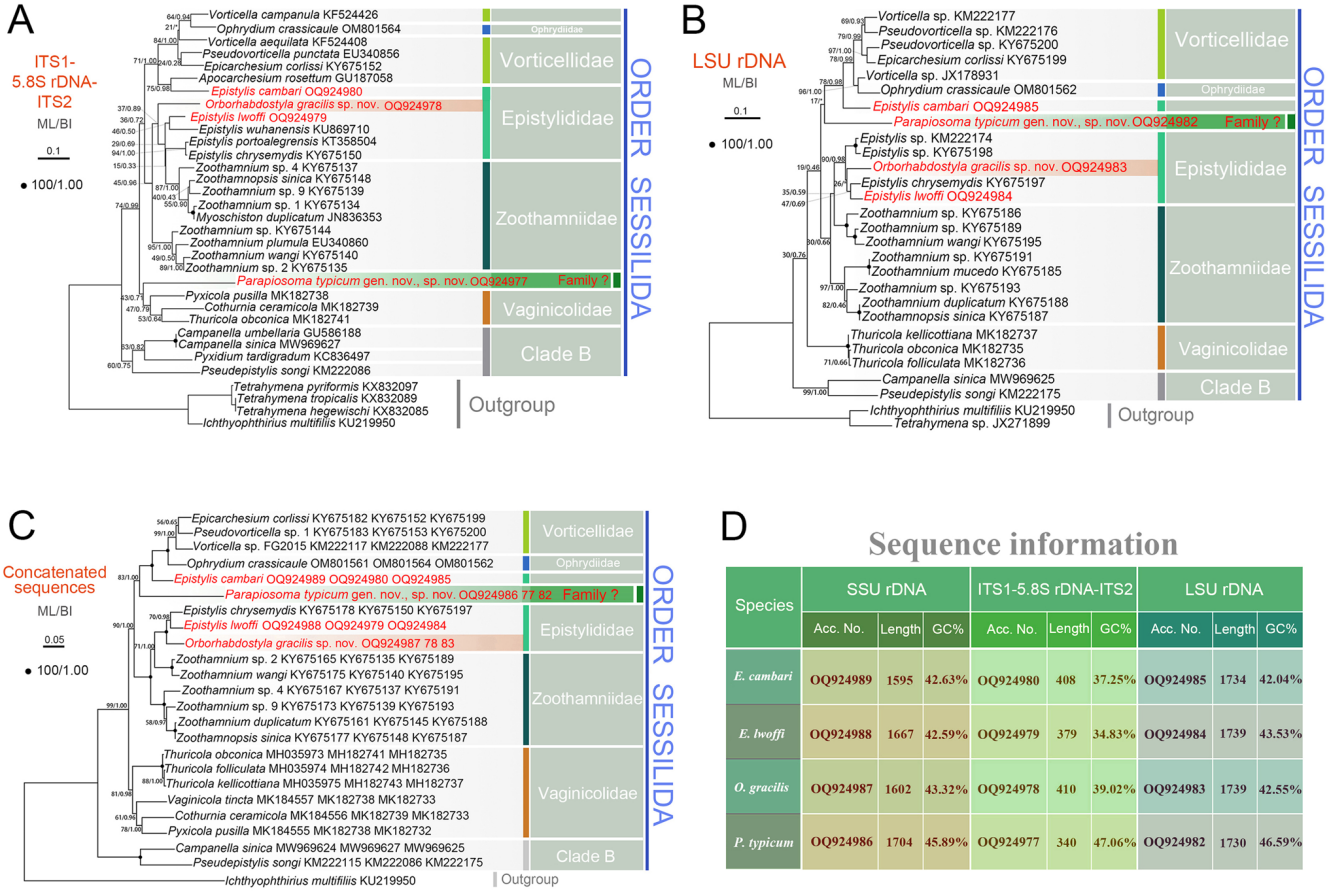
ML trees inferred from ITS1-5.8S rDNA-ITS2 (**A**), LSU rDNA (**B**), and concatenated sequences (**C**), and information for newly obtained sequences (**D**). Numbers near nodes denote maximum bootstrap values of ML out of 1000 replicates and posterior probabilities of Bayesian inference (BI). Asterisks indicate disagreements between ML and BI analyses. The scale bar indicates ten substitutions per 100 nucleotide positions in (**A**) and (**B**), five substitutions per 100 nucleotide positions in (**C**)

**Fig. 12:**
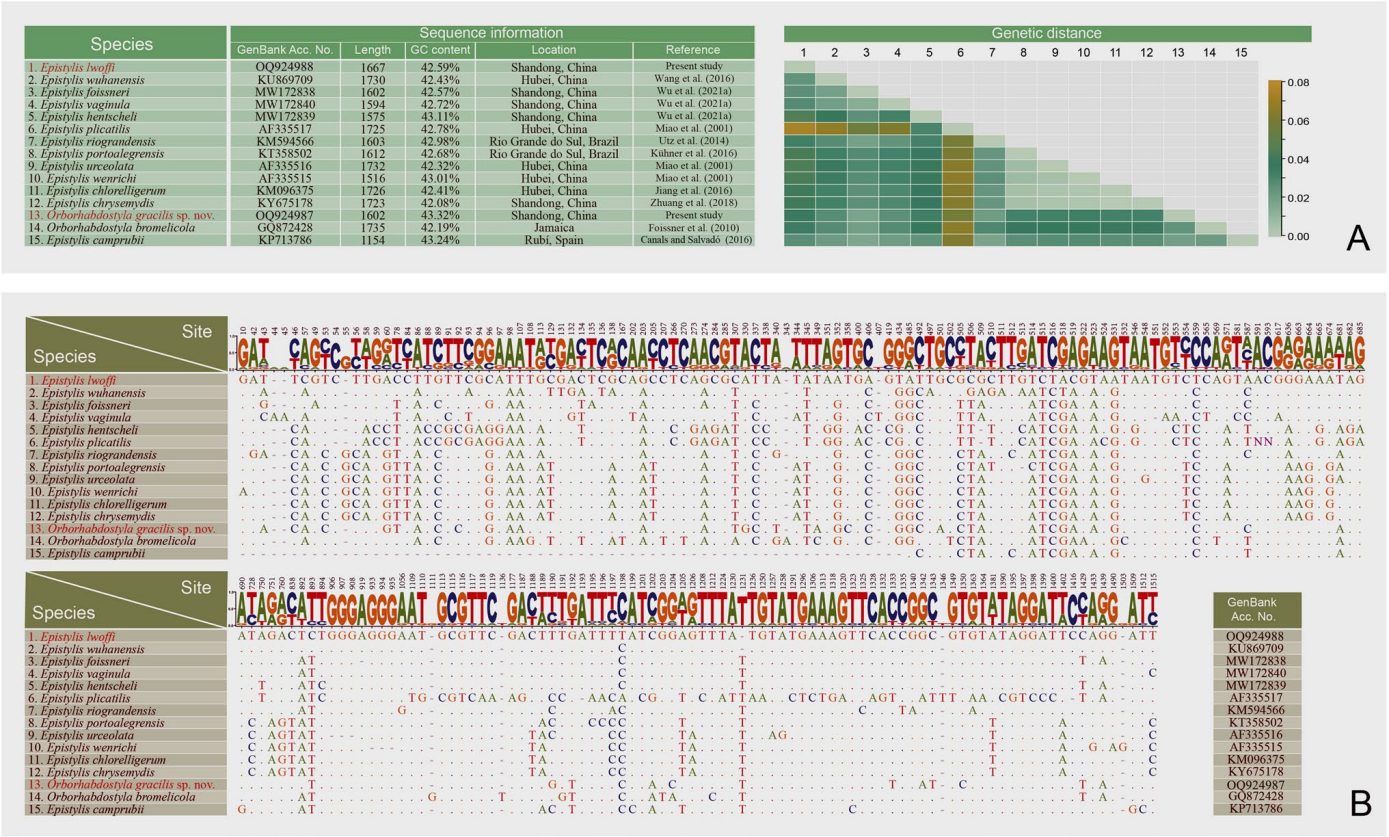
Nucleotide differences among *Epistylis lwoffi*, *Orborhabdostyla gracilis* sp. nov. and their closely related taxa (**A**, **B**) based on SSU rDNA sequences (sequence references: Canals and Salvadó i Cabré 2016; Foissner et al. 2010; Jiang et al. 2016; Kühner et al. 2016; Miao et al. 2001; Utz et al. 2014; Wang et al. 2017; Wu et al. 2021a; Zhuang et al. 2018). The numbers in the header indicate unmatched site positions

**Fig. 13:**
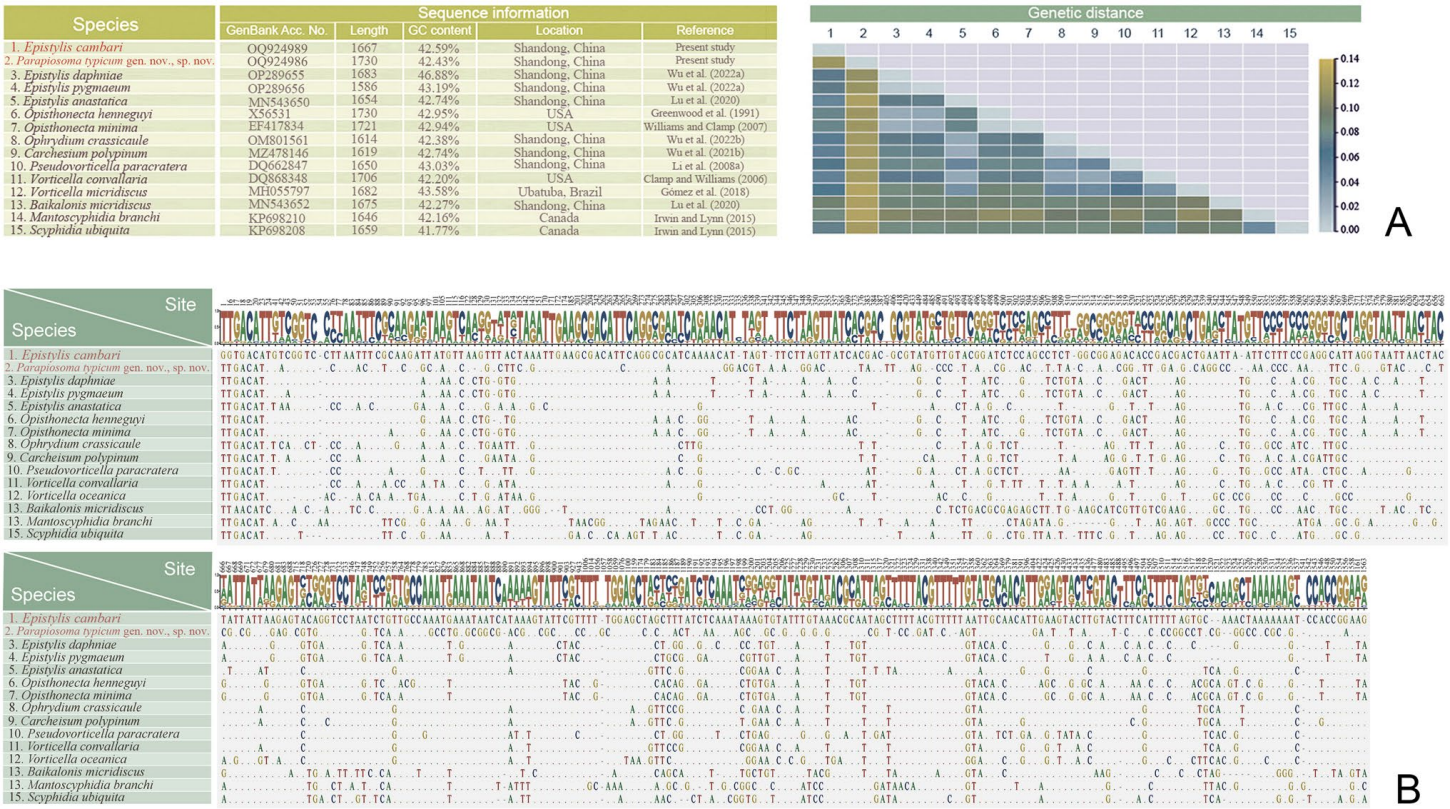
Nucleotide differences among *Epistylis cambari*, *Parapiosoma typicum* gen. nov., sp. nov. and their closely related taxa (**A**, **B**) based on SSU rDNA sequences (sequence references: Clamp and Williams 2006; Gómez et al. 2018; Greenwood et al. 1991; Irwin and Lynn 2015; Li et al. 2008a; Lu et al. 2020; Williams and Clamp 2007; Wu et al. 2021b, 2022a, b). The numbers in the header indicate unmatched site positions

**Sequence information.** The lengths, GC content and GenBank accession numbers of the 12 newly obtained sequences are summarized in Fig. 11D. The variable sites of the newly obtained sequences and closely related taxa are shown in Figs. 12 and 13.

**Phylogenetic analyses.** The topologies of the ML and BI trees are almost concordant for each gene, therefore only the ML trees, plus a concatenated tree, are presented.

In the SSU rDNA phylogenetic tree (Fig. 10), the members of family Zoothamniidae are separated into three clades (clades I–III). Clade II clusters with 13 *Epistylis* species (collectively called “core *Epistylis*”) and two *Orborhabdostyla* species (ML 85%, BI 1.00), forming a clade that is sister to clade I (ML 96%, BI 1.00). Clade III falls outside the assemblage comprising clade I, clade II, core *Epistylis*, and *Orborhabdostyla* (ML 95%, BI 1.00). *Epistylis lwoffi* groups with *E. wuhanensis* (ML 75%, BI 0.99) within the “core *Epistylis*” clade. The genus *Epistylis* is non-monophyletic with eight species not nesting within the “core *Epistylis*” clade. *Epistylis daphniae* and *E. pygmaeum* group with *Vorticellides astyliformis* and nest within a mixed clade (clade A) that includes both free-swimming and sessile species (ML 83%, BI 1.00). *Epistylis anastatica* clusters with two *Ophrydium* species and *Carchesium polypinum* (ML 28%, BI 0.40) to form a group that is sister to a clade comprising of two *Vorticella* species (ML 34%, BI 0.73). *Epistylis cambari* is located outside the assemblage formed by vorticellids and *Ophrydium* (ML 93%, BI 1.00). *Epistylis qingdaoensis*, *E. weishanensis*, and *E. elongata* nest within clade I. *Epistylis galea* groups with *Opercularia* (ML 99%, BI 1.00) within the basal clade (clade B) which also includes *Campanella* and *Pseudepistylis*. *Orborhabdostyla gracilis* sp. nov. groups with *Orborhabdostyla bromelicola* (ML 41%, BI 0.50) within clade II. *Parapiosoma typicum* sp. nov. is located outside the assemblage formed by clade A, Vorticellidae, and scyphidiids (ML 94%, BI 1.00).

In the ITS1-5.8S rDNA-ITS2 phylogenetic tree (Fig. 11A), *E. cambari* groups with *Apocarchesium rosettum* (ML 75%, BI 0.98), forming a clade that is sister to the assemblage formed by vorticellids and *Ophrydium*. *Epistylis lwoffi* groups with *E. wuhanensis* (ML 46%, BI 0.50), forming a clade that is sister to an assemblage formed by *Epistylis*, *Zoothamnium*, and *Myoschiston*. *Orborhabdostyla gracilis* sp. nov. is located outside the assemblage formed by *E. lwoffi* and *E. wuhanensis* and their sister clade mentioned above (ML 37%, BI 0.89). *Parapiosoma typicum* sp. nov. groups with a clade formed by three vaginicolids (ML 43%, BI 0.71).

In the LSU rDNA phylogenetic tree (Fig. 11B), *E. cambari* is located outside the assemblage formed by vorticellids and *Ophrydium* (ML 78%, BI 0.98). *Parapiosoma typicum* sp. nov. is located outside the assemblage formed by *E. cambari* and its sister clade mentioned above (ML 17%). *Epistylis lwoffi* groups with *E. chrysemydis* (ML 47%, BI 0.69), forming a sister clade of *Orborhabdostyla gracilis* sp. nov. (ML 26%).

In the concatenated tree (Fig. 11C), *E. cambari* is located outside the assemblage formed by vorticellids and *Ophrydium* (ML 100%, BI 1.00). *Parapiosoma typicum* sp. nov. is located outside the assemblage formed by *E. cambari* and its sister clade mentioned above (ML 83%, BI 1.00). *Epistylis lwoffi* groups with *E. chrysemydis* (ML 70%, BI 0.98), forming a sister clade of *Orborhabdostyla gracilis* sp. nov. (ML 100%, BI 1.00).

## Discussion

### Establishment of the genus *Parapiosoma* gen. nov.

Based on its non-contractile stalk and the everted peristomial lip, *Parapiosoma typicum* sp. nov. should belong to the family Epistylididae (Kahl 1935; Lynn 2008). Due to its characteristically obconical macronucleus, it most closely resembles *Apiosoma* Blanchard, 1885. However, *P. typicum* sp. nov. can form small colonies and therefore should not be included in the genus *Apiosoma*, members of which are solitary with the exception of *A. gasterostei* (Fauré-Fremiet, 1905) Scheubel, 1973. The solitary vs. colony lifestyle is a genus-level taxonomic character in Sessilida, such as the separation between *Gerda* (solitary) and *Ophrydium* (colonial) in Ophrydiidae. Thus, we establish a new genus *Parapiosoma* gen. nov. for *P. typicum* sp. nov., and assign *Parapiosoma gasterostei* (Fauré-Fremiet, 1905) comb. nov. (original combination: *Epistylis gasterostei* Fauré-Fremiet, 1905) to this new genus. *Parapiosoma gasterostei* (Fauré-Fremiet, 1905) comb. nov. was first reported by Fauré-Fremiet (1905) as “*Epistylis gasterostei*”. Precht (1935) transferred this species to the genus *Scyphidia*. Scheubel (1973) described another population and transferred this species to the genus *Apiosoma* based on the shape and position of its macronucleus. However, Scheubel (1973) reported that the stalk of *A. gasterostei* can be branched, i.e., this species is colony-forming, so we here transfer it to *Parapiosoma* gen. nov. Compared with *Parapiosoma typicum* sp. nov., *P*. *gasterostei* comb. nov. has a smaller zooid (40–70 × 22–34 μm vs. 70–90 × 30–35 μm) and a smooth (vs. transversely striated) stalk surface (Scheubel 1973).

### Family affiliations of *Parapiosoma* gen. nov. and *Apiosoma*

In all the present phylogenetic trees, *Parapiosoma typicum* sp. nov. forms a branch that is divergent from typical epistylidids and is sister to the Astylozoidae + Epistylididae + *Rhabdostyla commensalis* + Vorticellidae + Ophrydiidae + Scyphidiidae clade with high support (94% ML/1.00 BI) in the SSU rDNA tree. In the ITS1-5.8S rDNA-ITS2 tree, *P. typicum* sp. nov. is closely related to Vaginicolidae with weak support (43% ML/0.71 BI). In the LSU rDNA tree (ML analysis) and concatenated tree, *Parapiosoma typicum* sp. nov. is sister to the Vortcellidae + Ophrydiidae + *Epistylis cambari* clade. These findings indicate that *Parapiosoma typicum* sp. nov. may have evolved within a lineage composed of diverse clades and may represents a novel family.

*Parapiosoma* gen. nov. resembles *Apiosoma* in having goblet-shaped zooids, an obconical macronucleus, and a conspicuous trochal band located at or above the mid-body region. The genus *Apiosoma* was established by Blanchard (1885) and its validity was confirmed by Lom (1966). The taxonomic history of the genus *Apiosoma* was elaborated by Viljoen and Van As (1985). Kahl (1935) established the family Scyphidiidae, which is characterized by direct attachment to a substrate via the scopula, and assigned two genera, i.e., *Apiosoma* (*Glossatella* Bütschli, 1889) Blanchard, 1885 and *Scyphidia* Dujardin, 1841. *Apiosoma* has an obconical marconucleus located in the posterior region of the zooid whereas *Scyphidia* has a marconucleus that is not obconical in shape. The assignment of *Apiosoma* to the family Scyphidiidae was generally accepted (Banina 1968; Lom 1966; Scheubel 1973; Stiller 1971), however, its diagnosis has been revised several times. Consequently, Banina (1968) and Scheubel (1973) considered that all scyphidiids with a compact marconucleus should be assigned to *Apiosoma*, while Viljoen and Van As (1985) reported two species that possess a stalk, namely *A. caulata* Viljoen and Van As, 1985 and *A. micralesti* Viljoen and Van As, 1985. The diagnosis of *Apiosoma* is still unclear, not only because diagnoses proposed by different reasearchers conflict, but also because the distinction between *Apiosoma* and *Scyphidia* is blurred (Banina 1968; Kahl 1935; Lom 1966; Scheubel 1973; Shen and Gu 2016; Stiller 1971; Viljoen and Van As 1985). We suggest that the diagnosis of *Apiosoma* should be strictly based on the morphology of the type species *Apiosoma piscicola* Blanchard, 1885, which was described in detail by Blanchard (1885), and that an improved diagnosis should be supported by molecular systematics. Thus, molecular information of the type species and other species of *Apiosoma* are needed. In the classifications of Corliss (1979) and Lynn (2008), *Apiosoma* was assigned to the family Epistylididae. However, this assignment is questionable because the type species, *A. piscicola*, is stalkless whereas all other epistylidids are stalked (Banina 1968; Blanchard 1885; Blažeković-Dimovska and Stojanovski 2020; Li et al. 2008b, 2016; Lom 1966; Scheubel 1973; Viljoen and Van As 1985).

As mentioned above, *Parapiosoma* gen. nov. should be assigned to Epistylididae based on its morphology, especially its branched and non-contractile stalk. However, this placement is challenged by our phylogenetic analyses. Ever since Miao et al. (2004) provided the first molecular evidence to indicate that two epistylidids, namely *Epistylis galea* and *Campanella umbellaria*, belong to a separate lineage within the Sessilida, the systematics of the family Epistylididae has been in a state of flux. Most epistylidids form a monophyletic group that nests within clades of Zoothamniidae, whereas some epistylidid-like species are distributed in widely disparate clades in phylogenetic trees (Lu et al. 2020, 2023; Wu et al. 2022a; Zhuang et al. 2018). Unfortunately, although the molecular phylogeny of sessilids has been well investigated in recent years, corresponding morphological data and/or voucher specimens are not available for most sequences resulting in problems in determining the taxonomic placements of these epistylidid-like species. Additionally, molecular data for *Apiosoma* are lacking, therefore the systematic position of this genus and its phylogenetic relationship with *Parapiosoma* gen. nov. are unclear. Therefore, the establishment of a new family-level taxon for *Parapiosoma* gen. nov. is premature and should await a re-evaluation of the families Epistylididae and Scyphidiidae.

### Orborhabdostyla gracilis sp. nov.

The genus *Orborhabdostyla* was established by Foissner et al. (2010), and is characterized by its discoidal to ellipsoidal macronucleus (Foissner et al. 2010). However, according to the micrographs and illustrations in Foissner et al. (2010), the diagnosis of *Orborhabdostyla* should be revised based on it being a solitary epistylidid with a discoidal to ellipsoidal, flattened macronucleus and transverse silverlines. Hitherto, there were three valid species of *Orborhabdostyla*, namely *O. bromelicola* Foissner et al., 2010, *O. kahli* (Nenninger, 1948) Foissner et al., 2010, and *O. brevipes* (Claparède and Lachmann, 1858) Foissner et al., 2010, each of which can be morphologically separated from *Orborhabdostyla gracilis* sp. nov.

The type species of *Orborhabdostyla*,* O. Bromelicola*, was described in detail by Foissner et al. (2010). *Orborhabdostyla bromelicola* shares several morphological similarities with *O. gracilis* sp. nov. including zooid shape (both when extended and contracted), the single-layered peristomial lip, the shape of the peristomial disc, the position of the contractile vacuole, and the shape of the macronucleus. However, the zooid size differs significantly, i.e., 50–75 × 13–18 μm in vivo in *O. bromelicola* vs. 85–110 × 15–25 μm in vivo in *O. gracilis* sp. nov., and the oral ciliature also differs in that the abstomal ends of rows in P3 are clearly separated from each other in *O. bromelicola*, but are close together in *O. gracilis* sp. nov. (Foissner et al. 2010).

*Orborhabdostyla kahli* was described by Kahl (1935) under the name *Rhabdostyla* sp. Nenninger (1948) named it *Rhabdostyla kahli* and Foissner et al. (2010) transferred it to the genus *Orborhabdostyla*. *Orborhabdostyla kahli* differs from *O. gracilis* sp. nov. in having a smaller zooid (60–70 μm vs. 85–110 μm in length), a cylindrical (vs. conical) zooid shape, and a ventrally (vs. dorsally) located contratile vavuole (Foissner et al. 2010; Kahl 1935). In addation, Kahl (1935) described *O. kahli* as having a “peaked disk” and its peristomial disc is conical in Kahl’s illustration, which differs from the invariably flat peristomial disc of *O. gracilis* sp. nov.

*Orborhabdostyla brevipes* is a poorly known species with a very brief original description (Claparѐde and Lachmann 1858). Penard (1922) reported a population which he identified as *O. brevipes*. Kahl (1935), however, considered *O. brevipes* sensu Penard (1922) to be a different species because of its smaller zooid size (40–46 μm vs. 80–90 μm in vivo) and its vermiform (vs. ellipsoidal) macronucleus. Due to the original description of *O. brevipes* being very superficial, we agree with Foissner et al. (2010) who considered that the conspecificity of these historical populations cannot be confirmed until a population of *O. brevipes* collected from the original locality has been characterized using modern methods. In summary, *O. brevipes* can be distinguished from *O. gracilis* sp. nov. by its smaller zooid size in vivo (80–90 μm vs. 85–110 μm in length, mean 99.5 μm) and the cylindrical (vs. ovoidal) zooid shape when contracted (Claparѐde and Lachmann 1858; Foissner et al. 2010).

### *Epistylis cambari* Kellicott, 1885

*Epistylis cambari* was originally reported by Kellicott (1885) and is characterized by the following combination of features: (i) zooids about 50 μm long in vivo; (ii) single-layered peristomial lip; (iii) peristomial disc with a conical protuberance in its centre; (iv) contracted zooids with a snout-like projection at the anterior end; (v) stalk with transverse striations; (vi) widely spaced transverse pellicular striations; (vii) longitudinally oriented macronucleus; (viii) contractile vacuole located at the ventral wall of the infundibulum, beneath the peristomial lip. The Weishan population fits well with the original description except for the macronucleus orientation which is usually transversely oriented, but almost longitudinally oriented in some zooids (Fig. 7E). We believe the Weishan population is sufficiently similar to the type population of *E. cambari* for the two to be conspecific.

It is noteworthy that two other populations have also been identified as *E. cambari* since the original report, i.e., *E. cambari* sensu Matthes and Guhl (1973) and *E. cambari* sensu Lom and Puytorac (1994). *Epistylis cambari* sensu Matthes and Guhl (1973) has a contractile vacuole that is dorsally located at the same level as the peristomial lip, whereas in the original description, the contractile vacuole is ventrally located beneath the peristomial lip. In addition, the peristomial disc of *E. cambari* sensu Matthes and Guhl (1973) has only a small wart-like protuberance in its centre, unlike the original population which has conspicuously conical peristomial disc (Matthes and Guhl 1973). The peristomial disc of *E. cambari* sensu Lom and Puytorac (1994) does not have a clear conical protuberance. Furthermore, the inverted bell-shaped (vs. ellipsoidal in original population) zooid also conflict with the original population (Lom and Puytorac 1994). Thus, we believe the identities of these two populations need to be confirmed, which is consistent with the findings of Schödel (2018).

The most significant characters for separating *E. cambari* from its congeners are the peristomial disc with a conical protuberance, and the thick stalk with transverse striations on the surface and discontinuous rough longitudinal striations in the fibrillar matrix. Nevertheless, four similar species need to be compared with *E. cambari*, namely *E. crassicolis* Stein, 1854, *E. microdiscum* Stiller, 1963, *E. sommerae* Schödel, 1987 (= *E. thienemanni* Sommer, 1951), and *E. vasta* Sommer, 1951.

*Epistylis crassicolis* is a poorly defined species that is characterized by the presence of annular swellings beneath the branching points of the stalk (Kahl 1935; Stein 1854). Matthes and Guhl (1973) synonymized *E. ovalis* sensu Biegel (1954) with* E. crassicolis* based on this feature, which was accepted by Schödel (2018). In the original description of *E. crassicolis*, the contractile vacuole is dorsally located at the same level as the peristomial lip, but Matthes and Guhl (1973) and Biegel (1954) described the contractile vacuole as being ventrally located beneath the peristomial lip. Nevertheless, the original population and these other populations of *E. crassicolis* can be easily separated from *E. cambari* by their stalk with (vs. without) annular swellings beneath the branching points (Biegel 1954; Kahl 1935; Matthes and Guhl 1973; Schödel 2018; Stein 1854).

*Epistylis microdiscum* can be distinguished from *E. cambari* by its smaller zooids (45–55 × 30–35 μm vs. 55–70 × 25–40 μm in vivo), the position of the contractile vacuole (dorsally located vs. ventrally located), and the stalk without (vs. with) discontinuous rough longitudinal striations in the fibrillar matrix (Stiller 1963).

*Epistylis sommerae* differs from *E. cambari* by its smaller zooids (48–57 × 29–38 μm vs. 55–70 × 25– 40 μm in vivo), peristomial disc without (vs. with) a conical protuberance at its centre, and the greater number of silverline (22 vs. 14–16) between the trochal band and the scopula (Schödel 1987, 2018; Sommer 1951).

*Epistylis vasta* can be distinguished from *E. cambari* by its peristomial disc without (vs. with) a conical protuberance in its centre and its stalk with (vs. without) a hugely inflated second branch (Sommer 1951).

### *Epistylis lwoffi* Fauré-Fremiet, 1943

*Epistylis lwoffi* was first reported by Fauré-Fremiet (1943) and has been redescribed several times (Lom 1966; Lom and Vávra 1961; Scheubel 1973). In the original description, *E. lwoffi* is attached to the elongated basal part of the body of *Apiosoma* (*Glosatella*) *piscicola* by its stalk, the basal part of which is ring-shaped (Fauré-Fremiet 1943). However, the basal part of the stalk of *E. lwoffi* sensu Lom and Vávra (1961) is ring- or fork-shaped and is directly wedged into the mucous on the surface of the host, and other colonies can attach themselves to the main stalk. This arrangement is consistent with the findings of Scheubel (1973). Furthermore, in the original population, colonies may contain up to 10 zooids that are always located on one side of the stalk, whereas *E. lwoffi* sensu Lom and Vávra (1961) is solitary or forms colonies of only two zooids. Scheubel (1973) proposed that these are intraspecific differences resulting from environmental factors, especially food supply and temperature.

The Weishan population fits well with the following characters of *E. lwoffi*: (i) the inverted bell-shaped zooids; (ii) single-layered peristomial lip; (iii) C-shaped macronucleus that is usually transversely oriented; (iv) contractile vacuole dorsally located at the centre of the peristomial disc; (v) zooids always located at one side of the stalk; (vi) freshwater fish as the basiobiont. However, we failed to observe the ring- or fork-shaped basal end of the stalk because this part of the stalk was covered by large amounts of mucous. Nevertheless, the Weishan population of *E. lwoffi* generally appears together with *Parapiosoma typicum* sp. nov. (*E. lwoffi* often appears with *A. piscicola* in the original description) and several colonies are usually fixed in a position that looks like a bouquet, which is consistent with the original population. Lom (1964), however, reported that in *E. lwoffi*, the adstomal ends of polykineties1–3 terminates at the same level, which is not consistent with our observation. Nevertheless, based on the strong similarities of most features, we conclude that the Weishan population is conspecific with *E. lwoffi*.

Foissner and Schubert (1977) established the genus *Heteropolaria* into which they transferred *E. lwoffi*. *Heteropolaria* is characterized by the eccentric location of the scopula in the swarmer and the peculiar structure of the peristomial disc myoneme which branches off from the peristomial lip myoneme (Foissner et al. 1985). Prior to this, the location of the scopula was not generally acknowledged as a valuable diagnostic feature for sessilids. Furthermore, while we agree that the myoneme system is a taxonomically informative character, knowledge of the myoneme system is lacking for most peritrichs. Therefore, it is questionable whether these two characters can be used for genus definition. Foissner and Schubert (1977) assigned three species to *Heteropolaria*, i.e., *H. horizontalis* (type species), *H. colisarum*, and *H. lwoffi*. Unfortunately, molecular information is lacking for each. According to the present phylogenetic analyses, the Weishan population of *E. lwoffi* groups with *E. wuhanensis* within the “core *Epistylis*” clade. We therefore retain *E. lwoffi* in the genus *Epistylis* and consider the population of *H. lwoffi* reported by Foissner et al. (1983) to be a synonym of *E. lwoffi*. It is also noteworthy that Foissner et al. (1983) reported that the P3 of *E. lwoffi* is two-rowed (vs. three-rowed in the Weishan population), but we believe this may have been misinterpreted due to the rows of P3 often being obscured in stained specimens.

Two species with high similarity to *E. lwoffi* are *E. anastatica* (Linnaeus, 1767) Ehrenberg, 1830, and *E. plicatilis* Ehrenberg, 1831.

*Epistylis anastatica* can be easily distinguished from *E. lwoffi* by its ventrally (vs. dorsally) located contractile vacuole (Lu et al. 2020).

*Epistylis plicatilis* has been reported many times, but there are some conflicts among these previous reports, e. g., the terminal position of the adstomal end of P3 and the number of rows in P3, which have been discussed in Wu et al. (2021a). *Epistylis plicatilis* can be distinguished from *E. lwoffi* by its larger zooids (90–160 × 25–50 μm vs. 40–80 × 20–49 μm in vivo), clearly (vs. slightly) everted peristomial disc, regularly (vs. irregularly) dichotomously branched stalk, and the greater number of silverlines (176–206 vs. 70–92) between the peristome and the scopula (Foissner et al. 1992; Wu et al. 2021a).

### Phylogenetic analyses

According to our phylogenetic analyses, the genus *Epistylis* is non-monophyletic, which is consistent with previous studies (Wu et al. 2021a, 2022a; Zhuang et al. 2018). Recently, three *Epistylis* species nesting within clade A + Vorticellidae, i.e., *E. daphniae*, *E. pygmaeum*, and *E. anastatica*, were reported (Lu et al. 2020; Wu et al. 2022a). *Epistylis daphniae* and *E. pygmaeum* are small epibiotic species that share an unusual P3 pattern, i.e., the inner row is the longest row of P3 (Wu et al. 2022a). In the present phylogenetic trees, *E. cambari* is on a separate branch located outside the assemblage formed by vorticellids, ophrydiids, and *E. anastatica*. Unfortunately, we failed to find any morphological characteristics to support a close relationship between *E. cambari* and *E. anastatica*, although both are epibiotic species. The four *Epistylis* species mentioned above all group with species that have a stalk with a spasmoneme. Zhuang et al. (2018) proposed that *Zoothamnium*, a genus with a continuous spasmoneme that contracts in a “zig-zag” fashion, was ancestral to *Epistylis* s. str., which indirectly supports the phylogenetic position of these four *Epistylis* species. The ancestors of *Epistylis* were probably an assemblage consisting of different groups with a spasmoneme, including the genus *Zoothamnium*. The epibiotic lifestyle is an important selection factor that may play the key role in the evolution of epistylidids. Compared with species that live on inanimate substaters, epibiotic species would suffer more external stimuli, especially those attached to small fast-moving hosts, so they may have lost their spasmoneme and their stalk became non-contractile. Thus, they might represent a different evolutionary lineage to that of their congeners, which is consistent with the findings of Wang et al. (2022a) who suggested that lifestyle drives the diversity of aboral structures as well as diversification and evolution in peritrichs. *Epistylis lwoffi* groups with *E. wuhanensis* (ML 75%, BI 0.99). However, although both are epibiotic on freshwater fishes, we failed to find morphological support for this relationship. *Epistylis galea* (AF401527) invariably fell within clade B, but no morphological information is available for this sequence, so it needs to be re-investigated.

As expected, *Orborhabdostyla gracilis* sp. nov. groups with *O. bromelicola*, which is supported by their similar morphology, e.g., the zooid shape (both when extended and when contracted), the single-layered peristomial lip, the shape of the peristomial disc, the position of the contractile vacuole, and the shape of the macronucleus (Foissner et al. 2010). However, there are significant differences in their zooid size and oral ciliature (see above) and their SSU rDNA sequences differ by 40 base pairs.

*Parapiosoma* gen. nov. shares a high morphological similarity with *Apiosoma* (apart from the presence of a branched non-contractile stalk in the former) which suggests they are closely related. There is a possibility that these two genera may represent a distinct family, but molecular information of the genus *Apiosoma* is not available so a decision on this awaits further data. In the SSU rDNA tree, *P. typicum* sp. nov. does not nest within, nor is it sister to, the clade formed by *Scyphidia ubiquita* and *Mantoscyphidia branchi*. In the ITS1-5.8S rDNA-ITS2 tree, *P. typicum* sp. nov. sister to the Vaginicolidae, but there is no morphological support for this relationship. In the LSU rDNA and concatenated trees, the systematic position of *P. typicum* sp. nov. is broadly similar to that in the SSU rDNA tree. Therefore, more molecular data are needed to verify the phylogenetic position of *Parapiosoma*.

## Materials and methods

### Sample collection, observation, and identification

All the species were isolated from freshwater habitats in Lake Weishan Wetland, Shandong Province, China, during the period 2020 to 2021. *Epistylis lwoffi* and *Parapiosoma typicum* gen. nov., sp. nov. were isolated on 16 December 2020 from the fins of *Ctenopharyngodon idella* collected from an aquaculture pond (N34°44′21.44″; E117°09′33.80″) (Fig. 1A, B) when the water temperature was 8 °C. *Orborhabdostyla gracilis* sp. nov. was isolated on 18 June 2021 from a culture dish filled with water that contained mucous and scales collected from a fish stall in Weishan (N34°45′8.63″; E117°09′0.51″) (Fig. 1A, B) and maintained at 24 °C. *Epistylis cambari* was collected on 11 June 2021 from the maxilliped of *Procambarus clarkii* in Lake Weishan Wetland (N34°45′22.54″; E117°12′54.83″) (Fig. 1A, B).

Colonies were removed from their hosts or from the culture dish using acupuncture needles and collected with glass micropipettes. Specimens in vivo were observed using differential interference contrast microscopy at magnifications of 40 × to 1000 × with an Olympus BX 53 light microscope. The infraciliature was demonstrated using the protargol staining method (Wilbert 1975). The silverline system was revealed by the “dry” silver nitrate method (Foissner 2014). Terminology follows Warren (1986) and Foissner et al. (1992).

### DNA extraction, PCR amplification and sequencing

Genomic DNA was extracted from five zooids of each species using a DNeasy Blood & Tissue Kit (Qiagen, Hilden, Germany) following the manufacturer’s instruction. The primers and DNA polymerase used to amplify the SSU rDNA, ITS1-5.8S-ITS2, and LSU rDNA are the same as that in Wu et al. (2022b). PCR programs were designed according to Ma et al. (2021). PCR products were sequenced bidirectionally by the Tsingke Biological Technology Company (Qingdao, China).

### Phylogenetic analyses

Phylogenetic analyses were conducted with single-gene datasets of SSU rDNA, ITS1-5.8S rDNA-ITS2, and LSU rDNA separately, and with a concatenated dataset of all three genes or regions. Besides the 12 newly obtained sequences, other sequences were downloaded from the GenBank database, including sequences of 63 sessilids and three mobilids (*Trichodina sinonovaculae* FJ499386; *Trichodina ruditapicis* FJ499385; *Trichodina meretricis* FJ499387) for SSU rDNA; 25 sessilids and four hymenostomatians (*Tetrahymena tropicalis* KX832089; *Tetrahymena pyriformis* KX832097; *Tetrahymena hegewischi* KX832085; *Ichthyophthirius multifiliis* KU219950) for the ITS1-5.8S-ITS2 region; 22 sessilids and two hymenostomatians (*Tetrahymena* sp. JX271899; *Ichthyophthirius multifiliis* KU219950) for LSU rDNA. The mobilids and hymenostomatians mentioned above were used as out-group taxa.

Sequences were aligned and refined using MAFFT v.7 with default parameters (Katoh and Standley 2013). The two ends of the resulting alignment were trimmed in Gblocks v.0.91b (Castresana 2000; Talavera and Castresana 2007). The final lengths of the SSU rDNA, ITS1-5.8S rDNA-ITS2, and LSU rDNA were 1636 bp, 313 bp, and 1726 bp, respectively.

Maximum likelihood (ML) analyses with 1000 bootstrap replicates were computed using RAxML-HPC2 on XSEDE v.8.2.10 (Stamatakis 2014) on CIPRES Science Gateway (http://www.phylo.org) with GTRGAMMA + I model. Bayesian inference (BI) analyses were performed using MrBayes v.3.2.6 on XSEDE (Ronquist et al. 2012) on CIPRES Science Gateway with GTR + I + G model chosen by jModeltest v.2 (Darriba et al. 2012) according to the Akaike Information Criterion (AIC). Settings of Bayesian inference followed Wu et al. (2022b). Trees were visualized in MEGA X (Kumar et al. 2018). Classification is mainly according to Lynn (2008) and Gao et al. (2016).

### Nucleotide comparison of sequences

The rDNA sequences of the four present species and their related species were aligned by MAFFT v 7 via L-INS-I strategy (for GenBank accession numbers, see Figs. 12 and 13). The alignments were edited by BioEdit. Heatmaps were drawn by TBtools (Chen et al. 2020).

### Zoobank information

Publication LSID: urn:lsid:zoobank.org:pub:6CC84554-15A2-4CAE-A63E-67F7F2E51147.

Nomenclatural act LSIDs:

*Parapiosoma* gen. nov.: urn:lsid:zoobank.org:act:C6C45600-C97C-48F5-88E6-2E3621824936.

*Parapiosoma typicum* sp. nov.: urn:lsid:zoobank.org:act:43B9152F-531E-43AC-A11C-D5D028931C42.

*Orborhabdostyla gracilis* sp. nov.: urn:lsid:zoobank.org:act: 500C260A-3B28-4DCB-8A67-CDCB8379D508.

## Data Availability

All data generated or analyzed during this study are included in this published article.
